# KMT2C Loss Promotes NF2‐Wildtype Meningioma Progression and Ferroptosis Sensitivity via Epigenetic Repression of Hippo Signaling

**DOI:** 10.1002/advs.202522756

**Published:** 2026-02-05

**Authors:** Liuchao Zhang, Yangfan Ye, Wei Gu, Xinyue Wang, Nuo Chen, Qixin He, Lei Xu, Pengzhan Zhao, Guoqiang Fu, Guangyao Yuan, Wenqian Shi, Honglu Chao, Yiming Tu, Jing Ji

**Affiliations:** ^1^ Department of Neurosurgery The First Affiliated Hospital with Nanjing Medical University Nanjing Jiangsu P. R. China; ^2^ Neurovascular Center Changhai Hospital, Naval Medical University Shanghai P. R. China; ^3^ Department of Neurosurgery Division of Life Sciences and Medicine The First Affiliated Hospital of USTC University of Science and Technology of China Hefei Anhui P. R. China; ^4^ Department of Neurosurgery Northern Jiangsu People's Hospital Nanjing P. R. China; ^5^ Department of Neurosurgery Research Center of Clinical Medicine Affiliated Hospital of Nantong University, Medical School of Nantong University Nantong Jiangsu P. R. China; ^6^ Institute For Brain Tumors Jiangsu Key Lab of Cancer Biomarkers Prevention and Treatment Jiangsu Collaborative Innovation Center for Cancer Personalized Medicine Nanjing Medical University Nanjing Jiangsu P. R. China; ^7^ Department of Neurosurgery The Affiliated Kizilsu Kirghiz Autonomous Prefecture People's Hospital of Nanjing Medical University Artux Xinjiang P. R. China

**Keywords:** ferroptosis, histone acetylation, KMT2C, meningioma, NF2–Hippo signaling

## Abstract

High‐grade meningiomas remain clinically challenging due to their aggressive behavior and limited therapeutic options. Although mutations and dysregulation of KMT2 family members have been implicated in various cancers, their functional significance in meningioma remains unclear. While NF2 alterations are the most common drivers of meningioma pathogenesis, the mechanisms regulating NF2 transcription in NF2‐intact tumors are poorly understood. Here, we demonstrate that KMT2C expression is markedly reduced in high‐grade meningiomas and that loss of KMT2C promotes proliferation and invasion in NF2–wild‐type meningioma cells. Mechanistically, KMT2C deficiency suppresses NF2 transcription and inactivates Hippo signaling, leading to enhanced oncogenic activity and increased sensitivity to ferroptosis. Loss of KMT2C impairs the acetyltransferase activity of CBP/EP300, resulting in a global reduction of H3K27ac and transcriptional silencing of NF2. Pharmacological restoration of histone acetylation with the HDAC inhibitor Trichostatin A (TSA) effectively suppressed tumor growth. Collectively, our findings identify KMT2C as a key epigenetic regulator linking promoter histone acetylation, NF2–Hippo pathway activity, and ferroptosis susceptibility. These results provide mechanistic insights into high‐grade meningioma progression and highlight ferroptosis induction and epigenetic modulation as promising therapeutic strategies for NF2–wild‐type, KMT2C‐deficient meningiomas.

## Graphical abstract

In NF2–wild‐type meningiomas, loss of the epigenetic regulator KMT2C suppresses NF2 transcription and inactivates Hippo signaling, driving tumor progression and increasing ferroptosis sensitivity. Restoration of histone acetylation reverses these effects and inhibits tumor growth, identifying KMT2C as a key regulator linking epigenetic control, NF2–Hippo pathway activity, and ferroptosis vulnerability.

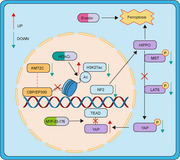

## 1 Introduction

Meningiomas are the most common primary intracranial tumors, accounting for 30%–40% of cases [1]. While most are benign (WHO grade I), approximately 20% present as atypical or malignant (grades II–III), grades II and III are considered high‐grade, exhibiting aggressive growth and frequent recurrence despite surgery and radiotherapy [2, 3]. The NF2 gene is a key driver in meningioma pathogenesis [4], and loss of NF2 disrupts the Hippo pathway, thereby promoting tumor progression and sensitizing cells to ferroptosis [5, 6, 7, 8]. Ferroptosis, a programmed cell death mechanism driven by iron accumulation and oxidative stress, has garnered significant attention in cancer therapeutic research [9]. Our prior work demonstrated that the NF2‐Hippo pathway inactivation sensitizes meningiomas to ferroptosis induction, highlighting its therapeutic relevance [10].

The KMT2 family of histone methyltransferases plays a pivotal role in enhancer regulation and has been recurrently implicated in cancer. For instance, translocations of the KMT2A gene are a common pathogenic mechanism in acute myeloid leukemia (AML), KMT2C is a frequent tumor suppressor gene in breast cancer, small cell lung cancer, and prostate cancer, and mutations in KMT2D are associated with follicular lymphoma and bladder cancer [11, 12]. KMT2C, one of the most frequently mutated genes in cancers including meningiomas [13, 14, 15, 16], encodes a histone methyltransferase that regulates enhancer activity through H3K4me1 and cooperates with CBP/EP300‐mediated H3K27ac deposition [17, 18, 19, 20, 21, 22]. Although KMT2C mutations are associated with poor prognosis in meningioma, its functional role in tumor biology remains unclear [23, 24, 25].

Here, we demonstrate that KMT2C loss diminishes H3K27ac levels, leading to NF2 transcriptional repression and Hippo pathway inactivation, thereby driving malignant progression. These findings establish KMT2C as a critical epigenetic regulator and potential therapeutic target in meningiomas.

## 2 Results

### 2.1 KMT2C Expression is Downregulated in High‐Grade Meningiomas

**FIGURE 1:**
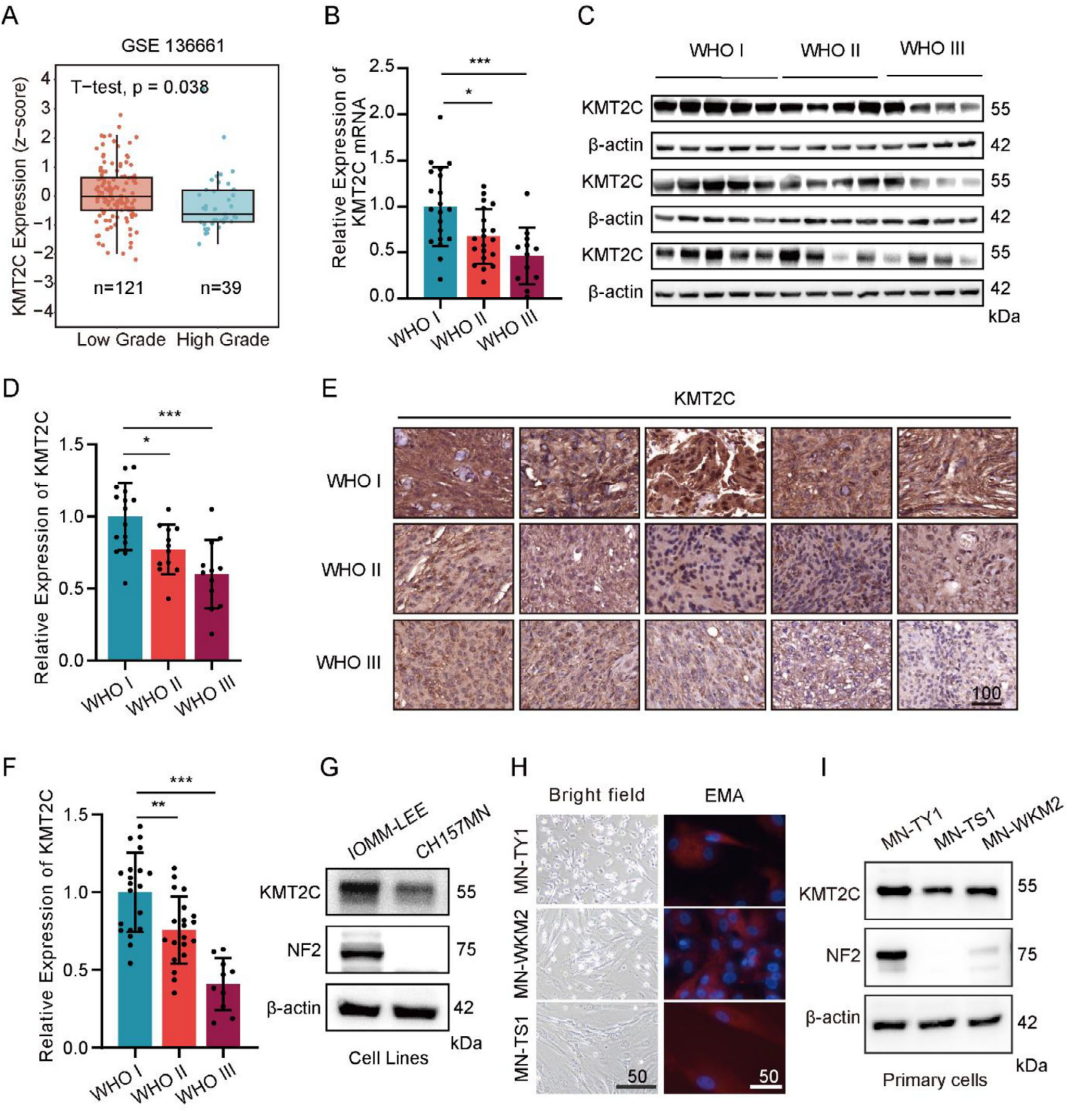
KMT2C expression is downregulated in high‐grade meningiomas. (A) mRNA expression levels of KMT2C in meningiomas from the GEO dataset GSE136661, normalized as Z‐scores and analyzed using an unpaired two‐tailed Student's *t*‐test. (B) Quantitative PCR analysis of KMT2C mRNA expression in clinical meningioma specimens (n = 20:20:10). (C) Western blot analysis of KMT2C protein levels in fresh‐frozen meningioma tissues, with β‐actin as a loading control. (D) Densitometric quantification of KMT2C protein expression shown in (C) (ImageJ; n = 15:12:12). (E) Representative immunohistochemistry (IHC) images showing KMT2C expression in meningioma tissues. Scale bar, 100 µm. (F) Quantification of IHC staining intensity using ImageJ (n = 20:20:10). (G) Western blot analysis of KMT2C protein expression in meningioma cell lines (IOMM‐Lee, CH157MN). (H) Representative light‐field and immunofluorescence images of primary meningioma cells stained for epithelial membrane antigen (EMA). Scale bar, 50 µm. (I) Western blot analysis of KMT2C protein expression in primary human meningioma cells. (G, I) Representative western blots are shown from n = 3 independent biological replicates. Data in (A) are presented as means ± SEM; data in (B), (D) and (F) are presented as means ± SD and analyzed using one‐way ANOVA. n.s for *p* > 0.05, *p < 0.05, **p < 0.01, ***p < 0.001.

Transcriptomic analysis of GEO dataset GSE136661 revealed significant downregulation of KMT2C in high‐grade meningiomas, while other KMT2 family members remained unchanged (Figure 1A; Figure S1A). qRT‐PCR of 50 tumors across WHO grades I–III confirmed a grade‐dependent reduction in KMT2C mRNA (Figure 1B), consistent with decreased protein levels detected by immunohistochemistry and immunoblotting (Figure 1C–F). These findings support a tumor‐suppressive role for KMT2C.

NF2 is the most frequently mutated gene in meningiomas, altered in approximately 50% of cases [26]. We analyzed two commonly used meningioma cell lines (IOMM‐Lee and CH157MN) and three primary meningioma cultures (MN‐TY1, MN‐WKM2, and MN‐TS1) to define NF2 and KMT2C status. Consistent with previous reports, IOMM‐Lee cells are NF2 wild type, whereas CH157MN cells harbor a deletion of the NF2 locus on chromosome 22q [10], accompanied by loss of NF2 protein expression (Figure 1G). Although DepMap data indicate a missense mutation in KMT2C in CH157MN cells, KMT2C protein expression was retained. Sanger sequencing identified an NF2 mutation in MN‐WKM2 cells (Figure S1B). In primary meningioma cells (Figure 1H), immunoblot analysis confirmed that KMT2C protein expression was preserved in MN‐TY1, MN‐WKM2, and MN‐TS1 cells, whereas NF2 protein expression was absent in MN‐WKM2 and MN‐TS1 cells (Figure 1I; Figure S1C).

### 2.2 KMT2C Deficiency Promotes Malignant Progression of NF2‐WT Meningiomas

**FIGURE 2:**
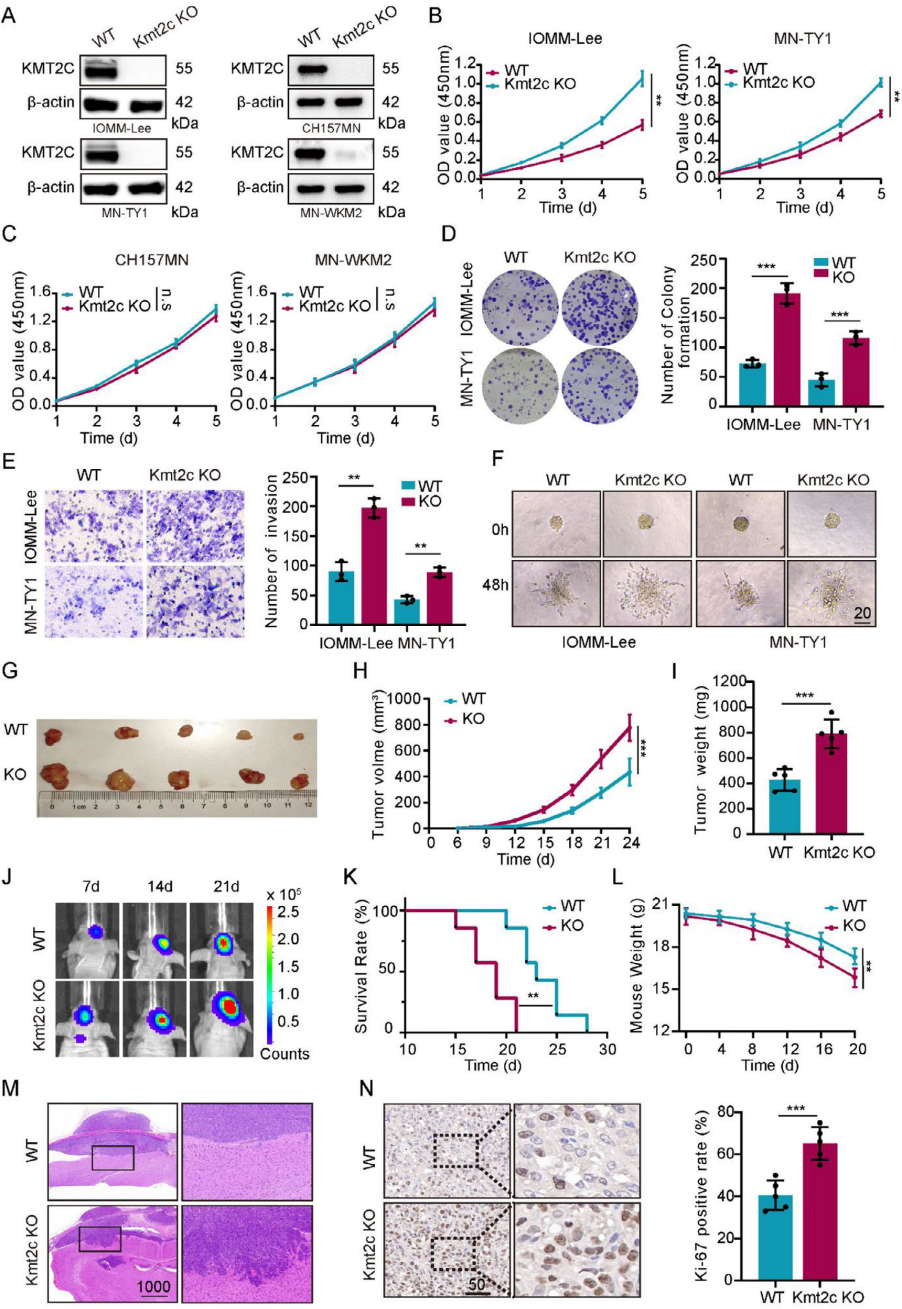
KMT2C loss promotes malignant progression of NF2‐WT meningiomas. (A) Western blot analysis of KMT2C expression in transfected meningioma cell lines and primary meningioma cells. (B, C) Cell viability determined by CCK‐8 assays in KMT2C‐WT and KMT2C‐KO IOMM‐Lee, MN‐TY1, CH157MN, and MN‐WKM2 cells (n = 3). (D) Representative images and quantification of colony formation in KMT2C‐WT and KMT2C‐KO IOMM‐Lee and MN‐TY1 cells (n = 3). (E) Representative transwell invasion assay images and quantification of invaded KMT2C‐WT and KMT2C‐KO IOMM‐Lee and MN‐TY1 cells after 24 h (n = 3). (F) Bright‐field images of tumorspheres formed by KMT2C‐WT and KMT2C‐KO cells in Matrigel‐based 3D culture. Scale bar, 20 µm. (G) Representative images of subcutaneous xenograft tumors derived from KMT2C‐WT and KMT2C‐KO IOMM‐Lee cells in nude mice. (H, I) Tumor volume and weight of subcutaneous xenografts (n = 5). (J) Representative bioluminescence imaging of intracranial xenografts at days 7, 14, and 21 after implantation of KMT2C‐WT and KMT2C‐KO IOMM‐Lee cells. (K) Kaplan–Meier survival analysis of mice (n = 7 per group). (L) Mouse body weight monitoring during the experiment (n = 7 per group). (M) Representative H&E‐stained brain sections from mice bearing KMT2C‐WT and KMT2C‐KO intracranial tumors. Scale bar, 1000 µm. (N) Immunohistochemistry for Ki‐67 in intracranial tumors. Scale bar, 50 µm (n = 5). (A) Representative western blots are shown from n = 3 independent biological replicates. Data are presented as means ± SD and analyzed using two‐way ANOVA with post hoc test or unpaired two‐tailed Student's *t*‐test. n.s for *p* > 0.05, *p < 0.05, **p < 0.01, ***p < 0.001.

CRISPR‐Cas9–mediated KMT2C knockout (KO) was successfully generated in both established and primary human meningioma models, including IOMM‐Lee, CH157MN, and patient‐derived MN‐TY1, MN‐WKM2, and MN‐TS1 cells. Immunoblotting confirmed complete loss of KMT2C protein expression in KO cells compared with isogenic wild‐type (WT) controls (Figure 2A; Figure S1E). In NF2‐wildtype (NF2‐WT) models, KMT2C deletion markedly enhanced tumor cell proliferation, as demonstrated by CCK‐8, EdU, and colony formation assays (Figure 2B,D; Figure S1D). KO cells produced more and larger colonies, consistent with increased DNA synthesis and growth. Transwell assays further showed significantly increased invasiveness in KMT2C‐KO IOMM‐Lee and MN‐TY1 cells (Figure 2E), while 3D Matrigel spheroid assays revealed extensive matrix penetration and irregular outgrowth compared to the compact morphology of WT spheroids (Figure 2F). By contrast, in NF2‐deficient CH157MN, MN‐WKM2, and MN‐TS1 cells, KMT2C loss did not enhance proliferation or invasion (Figure 2C; Figure S1F–I), indicating that KMT2C loss does not further enhance their malignant phenotypes. To further determine whether NF2 status influences KMT2C expression, immunohistochemical quantification was performed in clinical samples. These analyses revealed that NF2 loss did not alter KMT2C protein expression levels (Figure S1J,K).

In subcutaneous xenograft models, KMT2C‐WT IOMM‐Lee cells required higher inoculation doses to form tumors, whereas KMT2C‐KO cells readily produced rapidly enlarging masses. When equal numbers of WT and KO cells were injected, KMT2C loss significantly increased tumor growth rate and final tumor volume (Figure 2G–I). In orthotopic (intracranial) xenografts, bioluminescence imaging showed accelerated tumor expansion in KO‐bearing mice (Figure 2J), which exhibited shorter survival times (Figure 2K) and faster weight loss reflecting greater tumor burden (Figure 2L).

Histopathological examination revealed aggressive features in KMT2C‐KO tumors, including diffuse brain parenchymal invasion and architectural disruption (Figure 2M), whereas WT tumors remained circumscribed. Immunohistochemical staining confirmed significantly elevated Ki‐67 indices in KO tumors (Figure 2N), supporting enhanced proliferative capacity.

Collectively, these findings demonstrate that KMT2C acts as a critical tumor suppressor in NF2‐WT meningiomas, restraining proliferation and invasion both in vitro and in vivo.

### 2.3 KMT2C Deficiency Inactivates the Hippo Pathway in NF2‐WT Meningiomas

**FIGURE 3:**
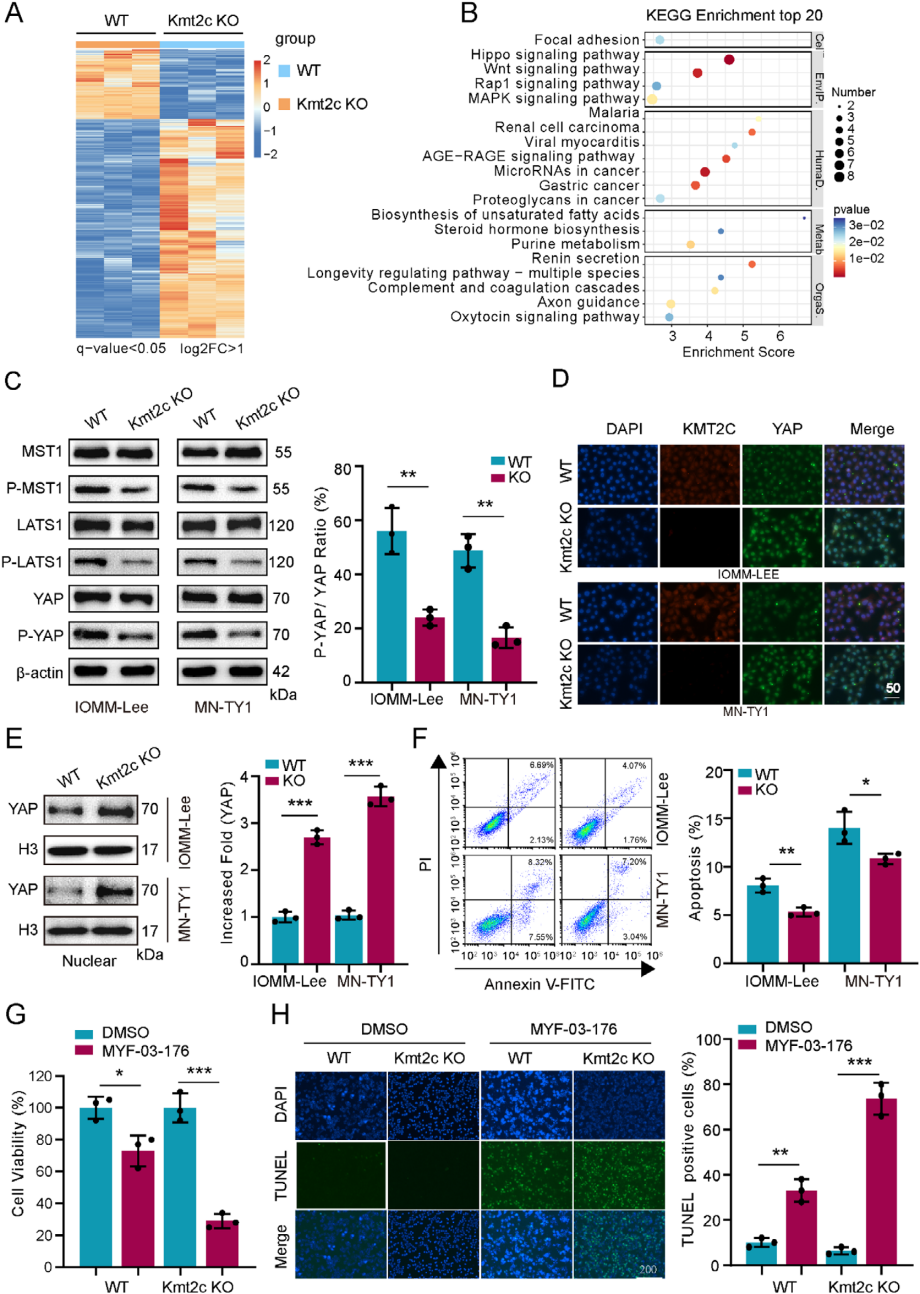
KMT2C loss suppresses Hippo signaling in NF2‐WT meningioma cells. (A) Heatmap showing significantly altered gene expression profiles in RNA‐seq analysis of KMT2C‐WT and KMT2C‐KO cells (n = 3 per group). (B) KEGG pathway enrichment analysis of downregulated genes in the KMT2C‐KO transcriptome. (C) Western blot analysis of YAP and phosphorylated YAP (p‐YAP) in KMT2C‐WT and KMT2C‐KO IOMM‐Lee and MN‐TY1 cells. Bar graphs (right) indicate the p‐YAP/YAP ratio normalized to β‐actin (n = 3). (D) Immunofluorescence staining of YAP in KMT2C‐WT and KMT2C‐KO IOMM‐Lee and MN‐TY1 cells, showing increased nuclear localization in KMT2C‐deficient cells. Scale bar, 50 µm. (E) Western blot of cytoplasmic and nuclear fractions showing enhanced nuclear YAP accumulation upon KMT2C loss. Bar graph shows densitometric quantification (ImageJ) (n = 3). (F) Flow cytometric analysis of apoptosis using Annexin V–FITC/PI staining. Representative plots and quantification of apoptotic cells (n = 3). (G) Cell viability assessed by CCK‐8 assay after 24 h treatment with MYF‐03‐176 (5 µm) in indicated groups (n = 3). (H) TUNEL staining (green) and DAPI counterstaining (blue) in MYF‐03‐176‐treated cells. Scale bar, 200 µm. Right: quantification of apoptotic index (n = 3). (C, E) Representative western blots are shown from n = 3 independent biological replicates. Data are presented as means ± SD and analyzed using one‐way ANOVA. n.s for *p* > 0.05, *p < 0.05, **p < 0.01, ***p < 0.001.

RNA‐seq of KMT2C‐knockout (KO) meningioma cells revealed significant enrichment of Hippo signaling alterations (Figure 3A,B; Figure S2A). Hippo pathway inactivation permits nuclear translocation of YAP/TAZ, where they activate transcription factors driving proliferation, development, and stemness. KO cells showed reduced YAP phosphorylation and decreased p‐YAP/YAP ratios (Figure 3C; Figure S2B), with enhanced nuclear YAP accumulation demonstrated through immunofluorescence and confirmed by Western blot analysis of nuclear fractions (Figure 3D,E). To determine whether YAP transcriptional activity was functionally activated, we examined the expression of canonical YAP/TEAD target genes, including CTGF, CYR61, ANKRD1, and AMOTL2. Notably, loss of KMT2C led to a consistent upregulation of these target genes at both the mRNA and protein levels (Figure S3C,D), providing further evidence that KMT2C deficiency results in functional inactivation of the Hippo pathway and consequent activation of YAP‐driven transcriptional programs in meningioma cells. Hippo pathway inactivation not only drives proliferation but also inhibits apoptosis [27]. Functionally, apoptosis was suppressed in KO cells (Figure 3F). *NF2*, the most frequently mutated gene in meningiomas, drives tumorigenesis through Hippo pathway inactivation and hyperactivation of YAP/TAZ, which promotes aberrant cell proliferation. In NF2‐mutant CH157MN, MN‐WKM2, and MN‐TS1 models, YAP phosphorylation and nuclear localization remained unchanged following KMT2C deletion (Figure S2E,F), supporting the conclusion that the tumor‐suppressive function of KMT2C requires intact NF2 signaling.

Hippo pathway inhibition has previously been shown to exhibit anti‐tumor efficacy in schwannomas and gliomas [28, 29]. MYF‐03‐176, a known Hippo pathway inhibitor that suppresses the nuclear interaction between YAP and TEAD, selectively impaired the survival of KO cells (Figure 3G; Figure S2G) and induced robust apoptosis (Figure 3H).

In summary, KMT2C knockout drives Hippo pathway dysregulation, leading to enhanced nuclear translocation of YAP, which promotes proliferation and inhibits apoptosis. The heightened apoptotic response of KMT2C‐deficient cells to MYF‐03‐176 underscores its potential therapeutic value for tumors harboring KMT2C loss.

### 2.4 KMT2C Deficiency Sensitizes NF2‐WT Meningioma Cells to Ferroptosis

**FIGURE 4:**
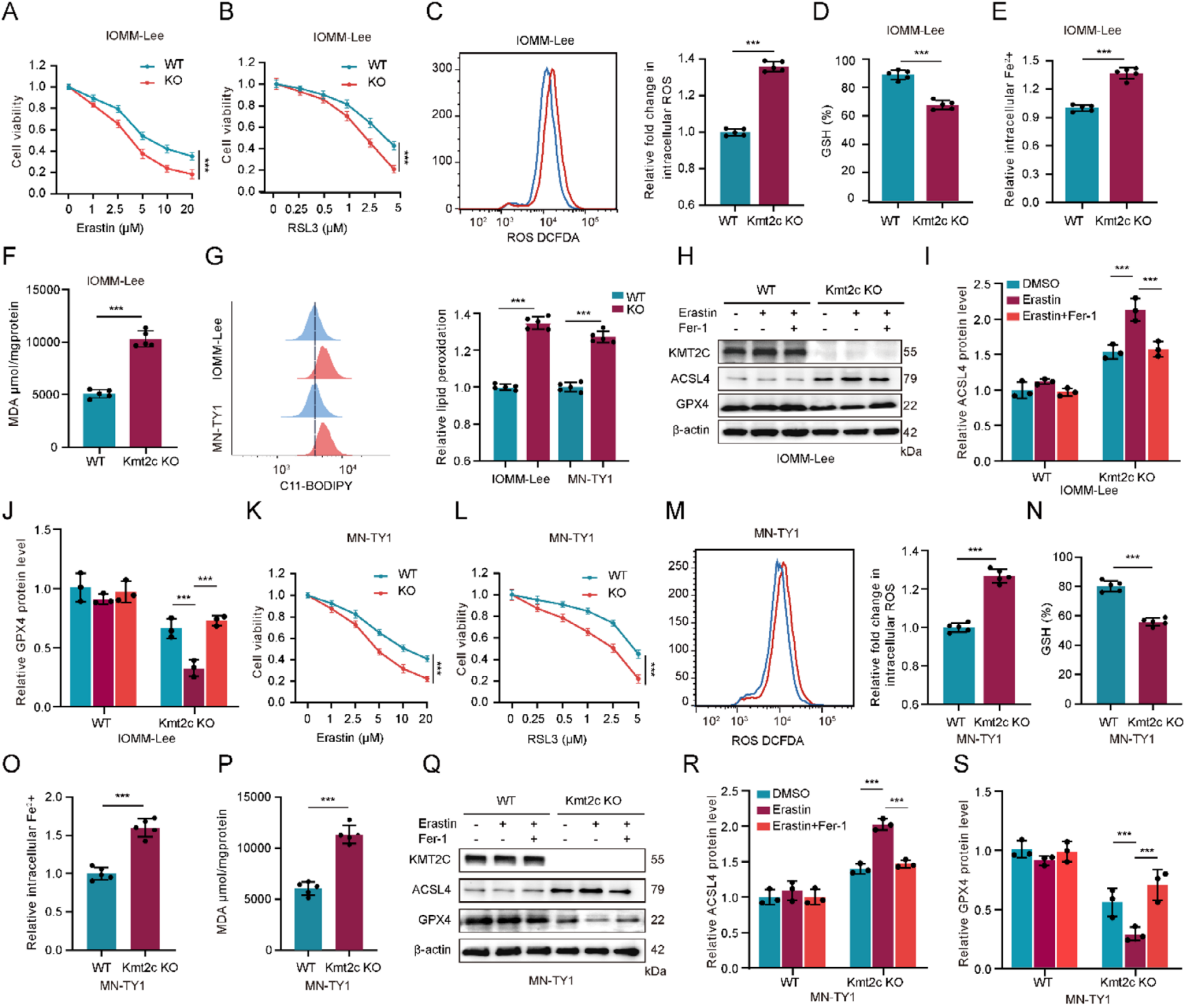
KMT2C loss sensitizes NF2‐WT meningioma cells to ferroptosis. (A, K) WT and KMT2C‐KO IOMM‐Lee and MN‐TY1 cells were treated with erastin (0–20 µm, 24 h), and cell viability was determined by CCK8 assay. (B, L) Cells were exposed to RSL3 (0–5 µm, 24 h), and viability was assessed as above. (C, M) Intracellular ROS levels were measured by DCFH‐DA staining and flow cytometry. (D, N) Intracellular GSH levels were quantified using GSH assay kits. (E, O) Cellular Fe^2^^+^ content was determined using FerroOrange fluorescence assay. (F, P) MDA levels were measured to evaluate lipid peroxidation. (G) Flow cytometric analysis for lipid peroxidation levels using C11‐BODIPY. (H, Q) WT and KMT2C‐KO IOMM‐Lee cells were treated with erastin (6 µm, 24 h) to induce ferroptosis, with or without Fer‐1 (3 µm, 24 h) as an inhibitor; western blot analysis was performed for KMT2C, ACSL4, and GPX4. (I, R) Densitometric quantification of ACSL4. (J, S) Densitometric quantification of GPX4. Data are presented as means ± SD. Data are analyzed by two‐way ANOVA with post hoc test and one‐way ANOVA. n.s for *p* > 0.05, *p < 0.05, **p < 0.01, ***p < 0.001.

Hippo signaling is closely linked to ferroptosis, a regulated cell death mechanism. Using ferroptosis inducers Erastin and RSL3, we observed that KMT2C knockout (KO) in IOMM‐Lee and MN‐TY1 cells led to a more pronounced reduction in cell viability with increasing drug concentrations (Figure 4A,B,K,L), indicating heightened ferroptosis sensitivity. Reactive oxygen species (ROS) play a central role in initiating and executing ferroptosis. We observed that KMT2C‐knockout (KO) meningioma cells exhibited elevated intracellular ROS levels (Figure 4C,M), indicative of heightened oxidative stress and ferroptosis susceptibility. Glutathione (GSH), a critical antioxidant, is utilized by glutathione peroxidase 4 (GPX4) to neutralize lipid peroxide. Erastin treatment markedly reduced GSH levels in KMT2C‐KO cells (Figure 4D,N), demonstrating impaired antioxidant capacity and enhanced oxidative damage. Concurrently, KMT2C‐deficient cells exhibited increased intracellular ferrous iron levels (Figure 4E,O) and higher concentrations of the lipid peroxidation product malondialdehyde (MDA) (Figure 4F,P). Consistently, BODIPY‐C11 staining revealed a marked elevation in lipid peroxidation (LPO) (Figure 4G), further confirming the enhanced ferroptotic vulnerability of KMT2C‐KO meningioma cells. E‐cadherin–mediated cell–cell adhesion suppresses ferroptosis through NF2–Hippo signaling, whereas disruption of this pathway upregulates ferroptosis drivers such as ACSL4 and transferrin receptor, while downregulating GPX4 [30, 31, 32, 33]. In KMT2C‐silenced cells, Erastin (6 µm) induced ACSL4 upregulation and GPX4 downregulation, effects reversed by the ferroptosis inhibitor Ferrostatin‐1 (Fer‐1) (Figure 4H–J,Q–S).

These results demonstrate that KMT2C deficiency amplifies ferroptosis sensitivity in meningiomas through dysregulation of oxidative stress homeostasis, iron metabolism, and antioxidant defense. This discovery provides novel insights into the role of KMT2C in meningioma pathobiology, specifically its modulation of ferroptosis pathways to influence tumor survival and treatment efficacy.

### 2.5 KMT2C Modulates Transcriptional Activity through Epigenetic Remodeling

**FIGURE 5:**
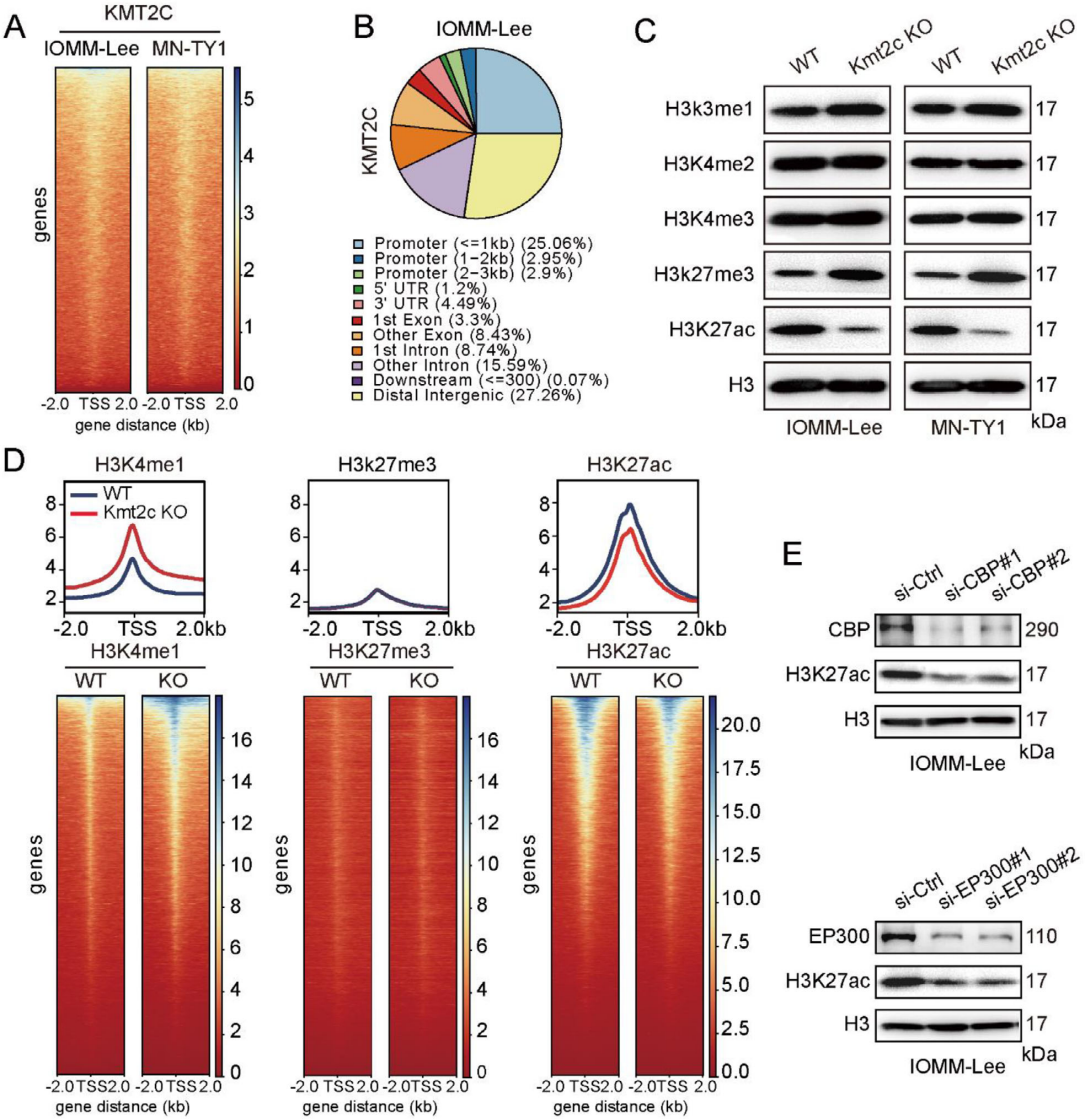
Loss of KMT2C reprograms the epigenomic landscape. (A) Heatmap showing CUT&Tag‐seq read density for KMT2C in WT IOMM‐Lee and MN‐TY1 cells. (B) Distribution of KMT2C‐binding peaks across different chromatin states. (C) Western blot analysis of histone modifications in KMT2C‐WT and KMT2C‐KO cells. (D) Average signal intensity profiles (top) and corresponding heatmaps (bottom) of CUT&Tag‐seq reads for H3K4me1, H3K27me3, and H3K27ac in WT and KMT2C‐KO IOMM‐Lee cells. (E) Western blot analysis of H3K27ac levels in IOMM‐Lee cells transfected with siRNA targeting CBP or EP300 for 72 h. (C, E) Representative western blots are shown from n = 3 independent biological replicates.

CUT&Tag‐seq in IOMM‐Lee and MN‐TY1 cells revealed preferential KMT2C occupancy at promoter regions, consistent with its transcriptional regulatory function (Figure 5A,B; Figure S3A). Loss of KMT2C altered histone landscapes, characterized by reduced H3K27ac and increased H3K27me3, as confirmed by immunoblotting (Figure 5C; Figure S3D). These antagonistic modifications indicate dynamic epigenetic reprogramming in meningiomas. Importantly, these epigenetic alterations were also recapitulated in clinical meningioma specimens. Immunohistochemical analysis demonstrated that tumors with low KMT2C expression exhibited decreased H3K27ac levels and increased H3K27me3 staining compared with tumors expressing high levels of KMT2C (Figure S3B), further supporting the clinical relevance of KMT2C‐mediated epigenetic remodeling observed in vitro.

Genome‐wide profiling further showed promoter‐centric changes in histone marks upon KMT2C deletion. TSS‐associated H3K4me1 peaks increased, whereas H3K27ac peaks decreased, while H3K27me3 enrichment remained globally elevated but unchanged at promoters (Figure 5D; Figure S3C), excluding its direct transcriptional role.

CBP and EP300 are the principal histone acetyltransferases responsible for catalyzing H3K27 acetylation in mammalian cells. To further examine whether reduced H3K27ac upon KMT2C loss is linked to CBP/EP300 activity, we performed siRNA‐mediated knockdown of CBP and EP300 in meningioma cells (Figure S3E). Depletion of CBP/EP300 resulted in a pronounced reduction in global H3K27ac levels, as confirmed by immunoblot analysis (Figure 5E; Figure S3F).

### 2.6 KMT2C is Required for CBP/EP300‐Mediated H3K27 Acetylation

**FIGURE 6:**
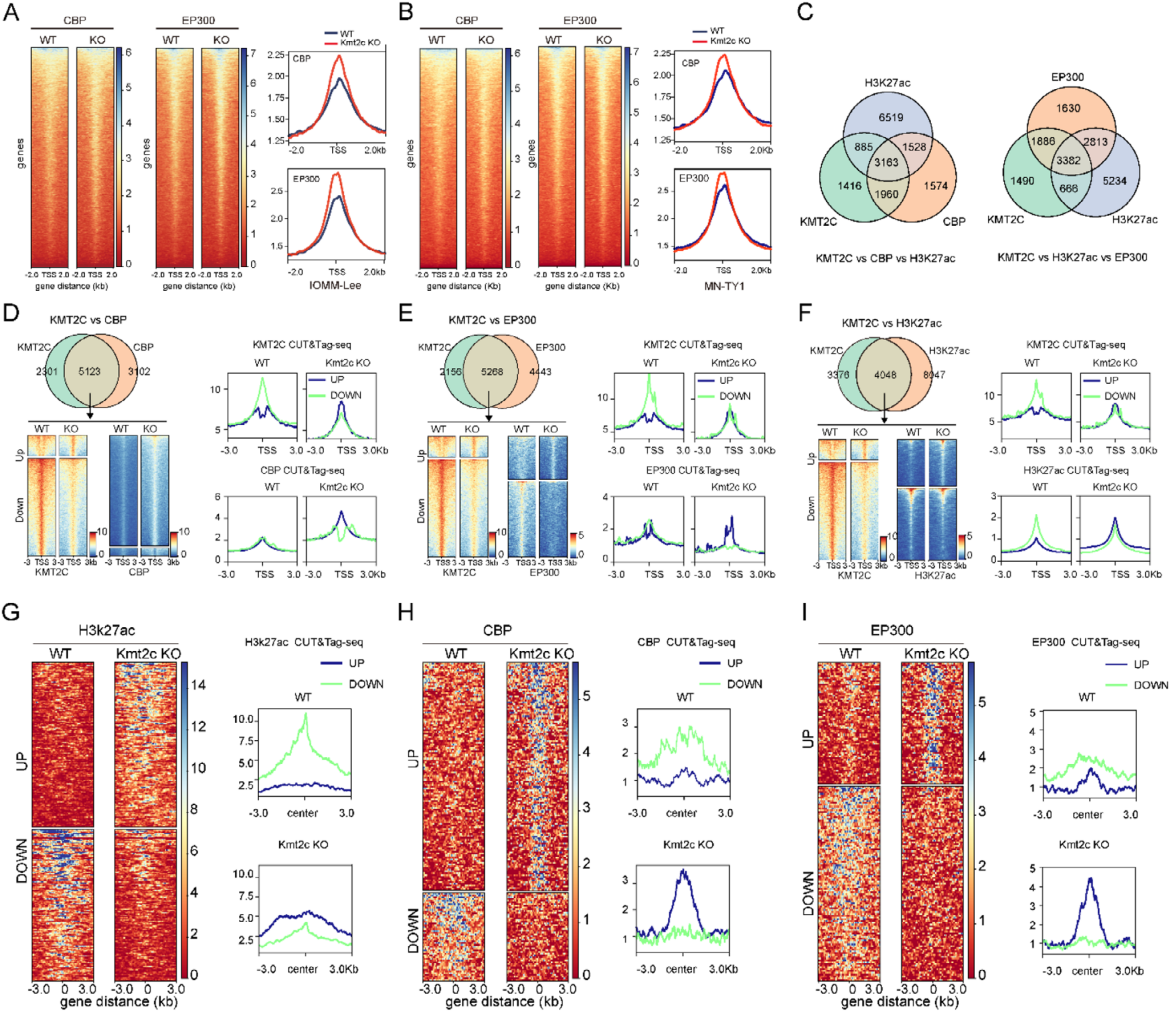
Loss of KMT2C impairs CBP/EP300 function. (A, B) Average signaling intensity curves and heatmap of CUT&Tag‐seq reads for CBP and EP300 in WT and KMT2C‐KO IOMM‐Lee and MN‐TY1 cells. (C) Venn diagram showing genome‐wide overlap among KMT2C, H3K27ac, and CBP/EP300 bound peaks from CUT&Tag‐seq analyses in human meningioma cells. (D–F) Heatmaps displaying differential CBP, EP300, and H3K27ac enrichment at KMT2C co‐occupied regions in WT and KMT2C‐KO IOMM‐Lee cells. (G–I) Aggregate plots showing changes in H3K27ac, CBP and EP300 signal intensity (±3 kb from peak centers) between WT and KMT2C‐KO cells.

CUT&Tag‐seq analysis revealed that CBP/EP300 occupancy at TSS regions was instead increased in KMT2C‐deficient cells (Figure 6A). Similar findings were replicated in MN‐TY1 cells, where KMT2C‐KO failed to reduce CBP/EP300‐TSS interactions (Figure 6B). These results indicate that although KMT2C loss profoundly alters histone modification patterns, it does not do so by disrupting CBP/EP300 binding to chromatin. KMT2C facilitates CBP/EP300 recruitment and H3K27ac enrichment by catalyzing H3K4me1 deposition at enhancers. KMT2C knockout disrupts this process, reducing H3K27ac levels [34]. Integrated CUT&Tag‐seq profiling in WT cells identified 3163 genomic regions co‐occupied by KMT2C, CBP, and H3K27ac (KMT2C+CBP+H3K27ac+), as well as 3382 regions co‐marked by KMT2C, EP300, and H3K27ac (KMT2C+EP300+H3K27ac+) (Figure 6C). Notably, 69% of KMT2C‐bound regions overlapped with CBP, 71% with EP300, and 55% with H3K27ac (Figure 6D–F), demonstrating their extensive co‐regulation at transcriptional elements. CUT&Tag‐seq analysis revealed a global redistribution of H3K27ac, CBP, and EP300 signals following KMT2C knockout (KO) (Figure 6G–I). Immunoblotting confirmed unchanged CBP/EP300 protein levels in KO cells (Figure S4A). Thus, KMT2C is dispensable for CBP/EP300 chromatin recruitment but essential for their acetyltransferase activity.

### 2.7 KMT2C Regulates NF2 Expression via H3K27ac in NF2‐WT Meningiomas

**FIGURE 7:**
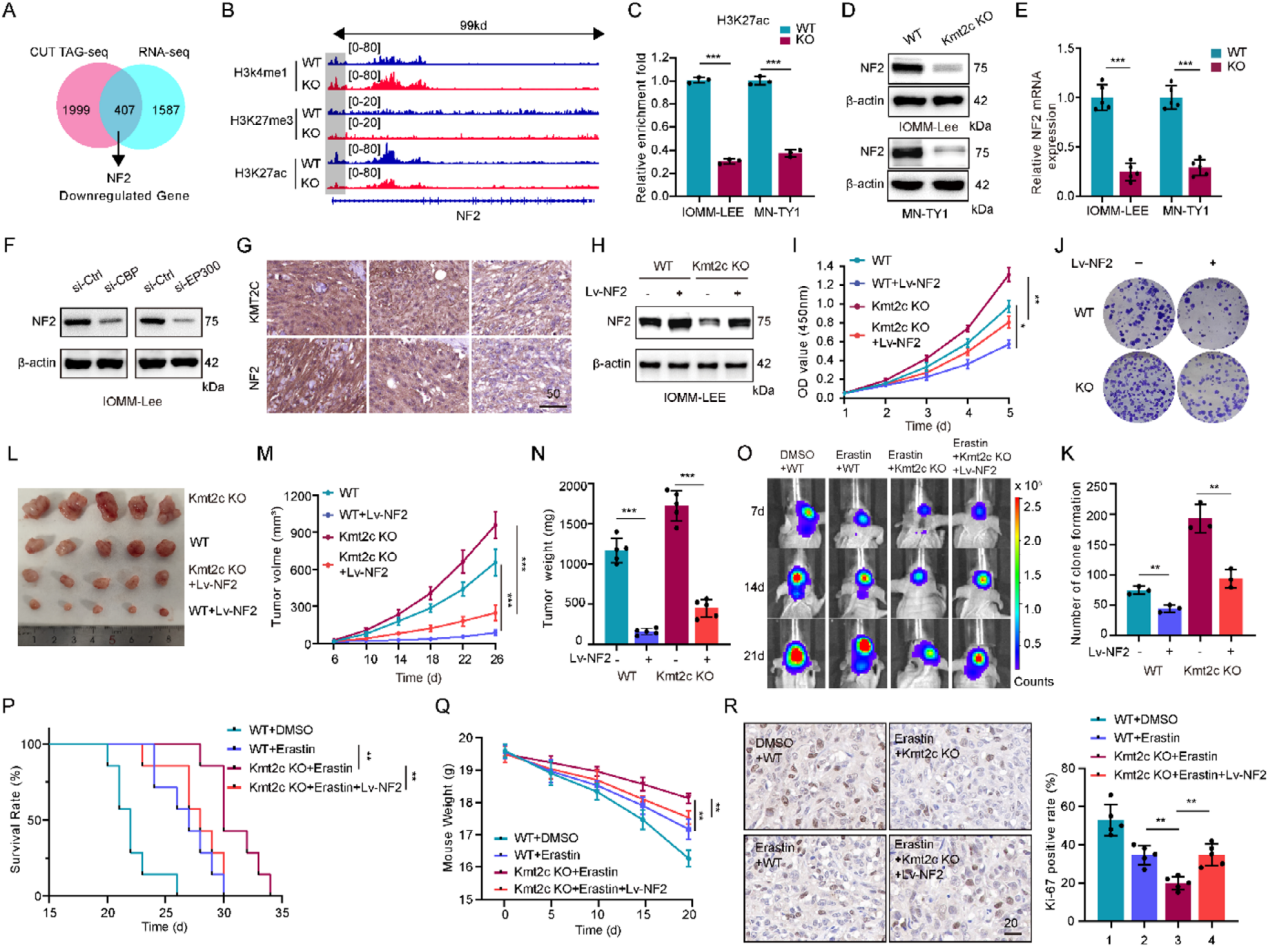
KMT2C regulates NF2 expression via H3K27ac, and NF2 suppresses tumor progression in KMT2C‐deficient meningiomas. (A) Venn diagram showing overlap between genes downregulated upon KMT2C loss (RNA‐seq) and those with reduced H3K27ac enrichment (CUT&Tag‐seq). (B) Genome browser tracks showing H3K4me1, H3K27me3, and H3K27ac peaks at the *NF2* locus in WT and KMT2C‐KO IOMM‐Lee cells. (C) CUT&Tag‐qPCR quantification of H3K27ac occupancy at the *NF2* promoter in WT and KMT2C‐KO IOMM‐Lee and MN‐TY1 cells (n = 3). (D, E) Western blot and qRT‐PCR analyses showing decreased NF2 protein and mRNA levels in KMT2C‐KO cells (n = 3). (F) Western blot analysis showing reduced NF2 expression following CBP or EP300 knockdown in IOMM‐Lee cells (72 h). (G) Representative IHC staining of KMT2C and NF2 in human meningioma specimens. Scale bar, 50 µm. (H) Western blot validation of NF2 overexpression in WT and KMT2C‐KO IOMM‐Lee cells. (I–K) CCK‐8 and colony formation assays showing that NF2 overexpression suppresses proliferation in WT and KMT2C‐KO IOMM‐Lee cells (n = 3). (L–N) Representative images, tumor growth curves, and weights of subcutaneous xenografts derived from indicated cells (n = 5). (O) Bioluminescence imaging of intracranial tumor burden in nude mice treated with Erastin, at days 7, 14, and 21 post‐implantation. (P) Kaplan–Meier survival analysis of mice bearing indicated tumors (n = 7 per group). (Q) Body weight monitoring during the experimental period (n = 7 per group). (R) IHC staining of Ki‐67 showing reduced proliferation upon Erastin treatment, reversed by NF2 overexpression. Scale bar, 20 µm (n = 5). (D, F, H) Representative western blots are shown from n = 3 independent biological replicates. Data are presented as means ± SD and analyzed by two‐way ANOVA with post hoc test or unpaired two‐tailed Student's t‐test. n.s for *p* > 0.05, *p < 0.05, **p < 0.01, ***p < 0.001.

Integrated RNA‐seq and CUT&Tag‐seq analyses revealed 336 genes upregulated through H3K4me1 gain and 407 genes downregulated due to H3K27ac loss, with the latter including NF2, a critical Hippo pathway regulator (Figure 7A; Figure S4B). CUT&Tag‐seq confirmed a pronounced reduction of H3K27ac at the NF2 promoter in KMT2C‐KO cells (Figure 7B; Figure S4C), further validated by CUT&Tag‐qPCR (Figure 7C). The increased CBP/EP300 signal at the NF2 promoter region in KO cells, observed through CUT&Tag sequencing on IGV (Figure S4D,F), was further validated by CUT&Tag‐qPCR (Figure S4E). Correspondingly, immunoblotting and qPCR demonstrated suppressed NF2 transcription and translation upon KMT2C loss (Figure 7D,E). CBP/EP300 knockdown similarly reduced NF2 expression via H3K27ac depletion (Figure 7F; Figure S4G), supporting its role in KMT2C‐mediated transcriptional regulation. Consistently, immunohistochemistry of clinical specimens revealed a positive correlation between KMT2C and NF2 expression (Figure 7G; Figure S5A). In addition, siRNA‐mediated knockdown of KMT2C in meningioma cells resulted in a marked reduction of both H3K27ac and NF2 expression (Figure S5B,C), phenocopying the effects observed in KMT2C knockout models. Functionally, CCK‐8 assays further demonstrated that partial depletion of KMT2C significantly enhanced meningioma cell proliferation (Figure S5D), indicating that even moderate reductions in KMT2C levels are sufficient to promote malignant growth.

CRISPR‐mediated NF2 knockout enhanced meningioma cell proliferation in vitro (Figure S5E–H). Functionally, NF2 overexpression inhibited proliferation in both WT and KO cells (CCK‐8 and colony formation assays, Figure 7H–K) and suppressed tumor growth in vivo, with more pronounced effects in KMT2C‐KO xenografts (Figure 7L–N; Figure S5I,M), confirming the tumor‐suppressive role of NF2 independent of KMT2C. Notably, NF2 overexpression abolished erastin‐induced ACSL4 elevation and GPX4 suppression in KMT2C‐KO cells (Figure S5J–L), confirming that KMT2C regulates ferroptosis through the NF2‐Hippo‐YAP axis. In an orthotopic meningioma model, Erastin markedly reduced intracranial tumor burden, with enhanced efficacy in KMT2C‐KO tumors; this effect was reversed by NF2 restoration (Figure 7O–Q). Consistently, Erastin's anti‐proliferative effects, reflected by decreased Ki‐67 staining, were abrogated upon NF2 overexpression (Figure 7R).

Together, these findings establish that KMT2C deficiency increases ferroptosis sensitivity through NF2–Hippo axis dysregulation, linking chromatin remodeling to oxidative stress and iron metabolism, and uncovering a therapeutic vulnerability in meningiomas.

### 2.8 Ferroptosis Sensitivity in KMT2C‐Deficient Meningiomas Depends on H3K27ac Status

Histone deacetylase (HDAC) inhibitors block H3K27ac deacetylation, thereby restoring acetylation levels and exerting anti‐tumor effects in meningiomas [35, 36, 37, 38]. Treatment with the HDAC inhibitor Trichostatin A (TSA) increased H3K27ac in both KMT2C‐WT and KO cells, reinstated NF2 mRNA and protein expression suppressed by KMT2C loss (Figure S6A–D), and markedly inhibited proliferation of KMT2C‐deficient cells (Figure S6E–H). In vivo, TSA treatment partially reversed the antitumor effect of Erastin in KMT2C‐KO meningiomas, leading to accelerated tumor growth and elevated Ki‐67 positivity (Figure S6I–L). These findings indicate that the ferroptosis susceptibility conferred by KMT2C loss is mediated through H3K27ac‐dependent mechanisms.

## 3 Discussion

Members of the COMPASS complex, KMT2C/D, are core chromatin remodelers that play crucial roles in regulating gene expression and cell differentiation [39]. Our study establishes KMT2C as an important tumor suppressor in meningiomas: loss of KMT2C accelerates tumor growth, suppresses apoptosis, enhances invasiveness, and promotes parenchymal infiltration, highlighting its broad functions in malignant progression.

The Hippo signaling pathway is frequently inactivated in various tumors, including mesothelioma and meningioma [40]. YAP/TAZ cooperate with TEAD transcription factors to drive the expression of pro‐tumorigenic genes [41]. In this study, KMT2C loss led to Hippo pathway inactivation, thereby promoting tumor growth and resistance to apoptosis. Hippo pathway inhibitors suppress YAP–TEAD–dependent transcriptional programs and thereby attenuate the expression of downstream oncogenic target genes. Accumulating evidence indicates that pharmacological inhibition of the Hippo–YAP axis exerts broad antitumor effects, including suppression of tumor cell proliferation, angiogenesis, and invasive behavior in multiple cancer types, such as pancreatic cancer and glioma [42]. MYF‐03‐176, a Hippo pathway inhibitor, preferentially induced apoptosis and inhibited proliferation in KMT2C‐deficient meningioma cells compared with KMT2C wild‐type controls, highlighting the potential of Hippo–YAP pathway inhibition as a precision therapeutic strategy for KMT2C‐deficient meningiomas.

Histone modification serves as a crucial epigenetic regulatory mechanism, influencing gene expression, DNA repair, and chromatin structure, thereby playing a pivotal role in tumor occurrence and development. At the epigenetic level, KMT2C deficiency resulted in increased H3K27me3 and decreased H3K27ac, consistent with previous findings. We further demonstrated that the HDAC inhibitor TSA could restore H3K27ac levels and suppress tumor growth in KMT2C‐deficient meningiomas. These findings suggest the therapeutic potential of HDAC inhibitors in meningiomas. However, H3K27me3 loss in meningiomas predicts shorter recurrence‐free survival, particularly in WHO grade II tumors [43]. Notably, HDACi‐mediated H3K27ac restoration may concomitantly reduce H3K27me3 levels, potentially exacerbating tumor aggressiveness [44]. Thus, further preclinical and clinical evidence is required to validate the therapeutic efficacy and safety of HDACi in meningioma patients, particularly in the context of H3K27me3 dynamics.

CBP and EP300, highly homologous transcriptional coactivators belonging to the KAT3 family of histone acetyltransferases (HATs), catalyze the acetylation of histones (e.g., H3K27) and non‐histone substrates (e.g., transcription factors) [45, 46]. Multiple studies demonstrate that CBP/EP300‐mediated H3K27ac deposition requires KMT2C/D. In adipocytes, KMT2C/D is essential for CBP/EP300 recruitment to enhancers and super‐enhancer (SE) formation, with KMT2C/D loss impairing CBP/EP300‐enhancer interactions [22]. In the present study, we show that KMT2C is required for efficient CBP/EP300‐dependent H3K27 acetylation in meningioma cells, while it is dispensable for CBP/EP300 chromatin binding. Despite preserved chromatin association of CBP/EP300, loss of KMT2C led to a marked reduction in H3K27ac levels. These findings support a critical role for KMT2C in facilitating CBP/EP300‐mediated transcriptional activation in meningiomas.

Notably, the precise mechanisms by which KMT2C modulates CBP/EP300‐dependent H3K27 acetylation remain incompletely understood. While some studies propose that KMT2C‐mediated H3K4me1 deposition creates a permissive chromatin environment for CBP/EP300 activity at regulatory elements, other reports suggest that KMT2C may regulate CBP/EP300 function through mechanisms at least partially independent of H3K4me1 [47, 48]. Together, these observations position KMT2C as a key epigenetic coordinator of CBP/EP300‐driven transcriptional programs through multifaceted regulatory mechanisms.

We further uncovered a critical functional link between KMT2C and NF2. Loss of KMT2C downregulated NF2 expression and selectively enhanced the malignant behavior of NF2–wild‐type meningiomas. In contrast, additional KMT2C loss in the NF2‐mutant background did not further exacerbate tumor progression, suggesting a potential epistatic interaction between KMT2C and NF2. Notably, KMT2C deficiency markedly sensitized meningioma cells to ferroptosis. Upon Erastin treatment, KMT2C‐deficient cells exhibited increased ACSL4 expression and decreased GPX4 levels—changes that were reversed by either Ferrostatin‐1 treatment or NF2 restoration—highlighting the dependence of ferroptosis susceptibility on NF2–Hippo pathway activity. Interestingly, the role of HDAC inhibition in regulating ferroptosis sensitivity appears to be context‐dependent: while HDAC inhibitors have been reported to synergize with ferroptosis inducers in colorectal cancer [35], our results revealed that HDAC inhibition suppressed ferroptosis in KMT2C‐deficient, NF2–wild‐type meningioma cells. Together, these findings indicate that KMT2C loss promotes ferroptosis susceptibility through inactivation of the NF2–Hippo–YAP axis, and that the interplay between histone acetylation and ferroptosis regulation may vary across tumor types. This highlights a potential therapeutic window for ferroptosis‐based or epigenetic interventions in aggressive meningiomas.

In conclusion, our study establishes the central epigenetic role of KMT2C in NF2‐wild‐type meningioma pathogenesis. By regulating histone acetylation, Hippo signaling, and ferroptosis, KMT2C loss drives malignant progression and uncovers novel therapeutic vulnerabilities, particularly highlighting the potential of HDAC inhibition, YAP–TEAD blockade, and ferroptosis induction as promising treatment strategies for meningiomas.

## 4 Experimental Section

### 4.1 Cell Lines and Cell Culture

IOMM‐Lee (RRID CVCL_5779) and CH157MN (RRID CVCL_5723) human meningioma cell lines were kindly given by Professor Wan's laboratory at H. Lee Moffitt Cancer Center in Tampa, FL. Both the meningioma cell lines were cultured in DMEM‐F12 medium (Gibco, 11320033) containing 10% Fetal Bovine Serum (FBS, Sigma) as a supplement.

### 4.2 Isolation of Primary Meningioma Cells

Primary meningioma cells were isolated from freshly resected tumor tissues. Tissues were washed three times in PBS containing antibiotics, minced into 1 mm^3^ fragments, and digested with 0.25% trypsin (1 mg/mL) at 37°C for 30 min with intermittent shaking. The reaction was quenched by adding serum‐supplemented medium. The suspension was filtered through a 70 µm strainer, centrifuged at 300 × *g* for 5 min, and treated with ACK lysis buffer to remove erythrocytes. Cell viability was assessed by trypan blue exclusion (>80% viability). Cells were plated at 1 × 10^6^ cells/mL, with the first medium change at 24 h to remove non‐adherent debris. Cultures were maintained with medium replacement every 48 h and passaged at 80% confluency using 0.25% trypsin (2 min at 37°C), followed by a 1:2 split ratio.

### 4.3 Antibodies and Chemicals

Antibodies and Chemicals used in this study are shown in Table S4.

### 4.4 Plasmids and Lentivirus Transduction

KMT2C‐knockout (KO) cell lines were generated via CRISPR/Cas9 editing using lentiviral vectors (Corues Biotechnology). Cells were transduced at 30%–50% confluency with lentiviral particles and Lipofectamine 2000. After 24 h incubation, fresh medium was added. Puromycin selection (2 µg/mL, 48 h) was followed by maintenance at 1 µg/mL for 7 days. NF2 constructs were engineered as described.

### 4.5 Western Blotting (WB)

Cells/tissues were lysed with SDS buffer containing protease/phosphatase inhibitors. Nuclear/cytoplasmic fractions were separated (Beyotime #P0027), resolved by SDS‐PAGE, and transferred to PVDF membranes. After blocking, membranes were incubated with primary antibodies (4°C, overnight), then HRP‐conjugated secondary antibodies. Signals were detected using chemiluminescence and quantified via ImageJ.

### 4.6 Immunohistochemistry (IHC)

FFPE meningioma sections were deparaffinized, antigen‐retrieved, and incubated with primary antibodies (anti‐KMT2C/NF2; 4°C, overnight), followed by HRP‐conjugated secondary antibodies. DAB and hematoxylin counterstaining were performed. Blinded evaluation was conducted by two independent investigators.

### 4.7 Tumorspheres Culture

Single‐cell suspensions were seeded in low‐adhesion plates with serum‐free medium. Spheroids formed within 5–7 Days were embedded in Matrigel (1:8 dilution) and imaged daily under phase‐contrast microscopy.

### 4.8 Subcutaneous Tumor Model

KMT2C‐KO or NF2‐KO IOMM‐Lee cells (2 × 10^6^ cells) were injected subcutaneously into 6‐week‐old nude mice. Erastin (15 mg/kg, i.p.) or vehicle was administered every other day from day 5. Tumor volume (V = 0.5 × length × width^2^) was measured every 3 days. Mice were euthanized on day 24; tumors were weighed and stored at −80°C.

### 4.9 Orthotopic Meningioma Survival Mode

Fluc‐expressing IOMM‐Lee Cells (4 × 10^5^ cells) were stereotactically implanted into the subdural space. tumor growth was monitored via IVIS bioluminescence imaging after D‐luciferin injection (10 mg/kg, i.p.) and euthanized until neurological signs occurred. Body weight and symptoms were recorded every 2 days. Blinded randomization ensured unbiased analysis.

### 4.10 TUNEL Staining

Cells were fixed, permeabilized, and stained using a TUNEL assay kit (Beyotime, C1088) following the manufacturer's instructions. Fluorescence images were acquired with a Leica DM5000B microscope, and TUNEL‐positive cells were manually counted.

### 4.11 Reactive Oxygen Species (ROS)

Intracellular ROS generation was assessed using the fluorescent probe DCFH‐DA (Beyotime, S0034S). Cells were incubated with 10 µm DCFH‐DA at 37°C for 20 min in the dark, followed by washing three times with PBS to remove residual dye. The fluorescence intensity was quantified using a microplate reader (Ex/Em = 488/525 nm, reflecting intracellular ROS levels.

### 4.12 Malondialdehyde (MDA)

Lipid peroxidation was quantified by measuring MDA using a commercial kit (Dojindo, M496). Cells were lysed, mixed with thiobarbituric acid reagent, and heated at 95°C for 15 min. After cooling to room temperature, samples were centrifuged, and the absorbance of the supernatant was measured at 532 nm. MDA concentrations were normalized to total protein levels.

### 4.13 Glutathione (GSH)

Total intracellular GSH was measured using a GSH detection kit (Beyotime, S0053). After cell lysis and deproteinization, the supernatant was mixed with DTNB reagent, and absorbance was recorded at 412 nm. GSH content was calculated according to the standard curve and normalized to protein concentration.

### 4.14 Intracellular Fe^2^^+^

The labile Fe^2^^+^ pool was visualized using FerroOrange (Dojindo). Cells were incubated with 1 µm FerroOrange for 30 min at 37°C in the dark, washed with HBSS, and immediately quantified by a microplate reader (Ex/Em = 543/580 nm).

### 4.15 CUT&Tag Sequencing

The CUT&Tag Assay (Vazyme #TD904) was performed using 1 × 10^5^ cells permeabilized with digitonin buffer and incubated with primary antibodies (KMT2C: Thermo fisher 6D1B9; H3K27me3/H3K27ac: CST #9733/#8173; 1:100) overnight at 4°C. Secondary antibodies (Vazyme #Ab207‐01/#Ab208‐01) were added for 60 min. Tagmentation, DNA purification, and library preparation (Vazyme #TD202‐00) followed kit protocols. Libraries were sequenced on illumina NovaSeq 6000 (150‐bp reads per sample).

### 4.16 CUT&Tag‐qPCR Validation

qPCR (SYBR Green Master Mix, Vazyme) was performed on purified CUT&Tag DNA using target‐specific primers (e.g., NF2 promoter) normalized to input. Antibodies included H3K27ac (CST #8173, 1:100), CBP (GeneTex #GTX101249, 1:100), EP300 (ABclonal #A13016, 1:50). Enrichment fold‐changes were calculated via 2−^ΔΔct^ with IgG controls.

### 4.17 RNA Sequencing Experimental Method

Total RNA was extracted using the TRIzol reagent (Invitrogen, CA, USA) according to the manufacturer's protocol. RNA purity and quantification were evaluated using the NanoDrop 2000 spectrophotometer (Thermo Scientific, USA). RNA integrity was assessed using the Agilent 2100 Bioanalyzer (Agilent Technologies, Santa Clara, CA, USA). Then the libraries were constructed using VAHTS Universal V6 RNA‐seq Library Prep Kit according to the manufacturer's instructions. The transcriptome sequencing and analysis were conducted by OE Biotech Co., Ltd. (Shanghai, China).

### 4.18 Statistical Analysis

Statistical analyses were performed using GraphPad Prism 9.0 (GraphPad Software, USA). Data are presented as mean ± standard deviation (SD) or mean ± standard error of the mean (SEM) derived from at least three independent experiments. Differences between groups were analyzed using Student's *t*‐test (two‐tailed), one‐way ANOVA, or two‐way ANOVA with appropriate post‐hoc corrections (e.g., Tukey's or Sidak's test). Survival curves were compared via the log‐rank test for Kaplan‐Meier analyses. *p*‐value < 0.05 was considered statistically significant.

## Author Contributions

Conception and design: J.J. and Y.T.; Data acquisition, analysis, and interpretation: L.Z., N.C., and Q.H.; Investigation: L.Z., W.G., L.X., P.Z., and W.S.; Acquisition of patient specimens: L.Z., G.F., and G.Y.; Article drafting and revising: L.Z., H.C., and Y.T.; Article writing: L.Z., Y.Y., F.C., and X.W.; All authors approved the final version of the manuscript.

## Funding

This work was supported by the National Natural Science Foundation of China [Grant Nos. 82120108018 to J.J., 82303835 to Y.T.], the National Key Research and Development Program of China [Grant No. 2021YFA1101802‐2 to J.J.], the Priority Academic Program Development of Jiangsu Higher Education Institutions [Grant Nos. JX10231803, JX10231804 to J.J.], the Xinjiang Uygur Autonomous Region Tianshan Innovation Team Plan Item [Grant No. 2024D14012 to J.J.], the China Postdoctoral Science Foundation [Grant No. 2021M701495 to Y.T.], the Fostering Fund of the First Affiliated Hospital of Nanjing Medical University [Grant No. PY2025030 to Y.Y.].

## Ethics Statement

Tumor sample collection was approved by the Institutional Review Board of the First Affiliated Hospital with Nanjing Medical University (JSSRY‐KY16‐022), with written informed consent from all patients. This study involved animal experiments that were approved by the Ethics Review Committee of Nanjing Medical University (IACUC‐2205076). All methods were performed in accordance with the relevant guidelines and regulations.

## Conflicts of Interest

The authors declare no conflicts of interest.

## Supporting information




**Supporting File 1**: advs74199‐sup‐0001‐SuppMat.pdf.

**Supporting File 2**: advs74199‐sup‐0002‐Data.zip.

## Data Availability

The data that support the findings of this study are openly available in [Gene Expression Omnibus (GEO)] at [http://www.ncbi.nlm.nih.gov/geo/], reference number [297610].
