# Supplementary material for: KMT2C Loss Promotes NF2‐Wildtype Meningioma Progression and Ferroptosis Sensitivity via Epigenetic Repression of Hippo Signaling
Source: Adv Sci (Weinh). 2026 Feb 5;13(20):e22756. doi: 10.1002/advs.202522756 (PMC13067829; doi:10.1002/advs.202522756)
Supplement: Supplementary file 1 — Supporting File 1: advs74199‐sup‐0001‐SuppMat.pdf. [file ADVS-13-e22756-s001.pdf]

# Supporting Information

**KMT2C loss promotes NF2-wildtype meningioma progression and ferroptosis sensitivity via epigenetic repression of Hippo signaling**

*Liuchao Zhang[1,#], Yangfan Ye[1,#], Wei Gu[1,#], Xinyue Wang[1], Nuo Chen[1], Qixin He[1], Lei Xu[4], Pengzhan Zhao[1,5], Guoqiang Fu[6], Guangyao Yuan[7], Wenqian Shi[1], Honglu Chao[1], Yiming Tu[1,✉], Jing Ji[1,2,3,✉]*

[#] These authors contributed equally to this work.

[1] Department of Neurosurgery, The First Affiliated Hospital with Nanjing Medical University, Nanjing, 210029, Jiangsu, China.

[2] Institute for Brain Tumors, Jiangsu Key Lab of Cancer Biomarkers, Prevention and Treatment, Jiangsu Collaborative Innovation Center for Cancer Personalized Medicine, Nanjing Medical University, Nanjing, 211166, Jiangsu, China.

[3] Department of Neurosurgery, The Affiliated Kizilsu Kirghiz Autonomous Prefecture People's Hospital of Nanjing Medical University, Artux, Xinjiang, China.

[4] Neurovascular Center, Changhai Hospital, Naval Medical University, Shanghai, China.

[5] Department of Neurosurgery, The First Affiliated Hospital of USTC, Division of Life Sciences and Medicine, University of Science and Technology of China, Hefei, Anhui Province, PR China.

[6] Department of Neurosurgery, Northern Jiangsu People's Hospital.

[7] Department of Neurosurgery, Research Center of Clinical Medicine, Affiliated Hospital of Nantong University, Medical School of Nantong University, Nantong,

Jiangsu 226001, China.

✉ **Corresponding author:**

Jing Ji, MD, Ph.D. Department of Neurosurgery, The First Affiliated Hospital with Nanjing Medical University, Nanjing 210029, Jiangsu, China. Phone number: +8613912976265; E-mail: jijing@njmu.edu.cn

Yiming Tu, MD, Ph.D. Department of Neurosurgery, The First Affiliated Hospital with Nanjing Medical University, Nanjing 210029, Jiangsu, China. Phone number: +8615895903128; E-mail: yimingtu2018@njmu.edu.cn

**Supplementary Table 1**. Clinical data of enrolled patients

| Clinical data | n = 50 |
|---|---|
| Sex | |
| Male | 28 |
| Female | 22 |
| Age | |
| Mean | 56.8 ± 13.06 years |
| Median | 56 years |
| Range | 30 - 82 years |
| WHO Grade | |
| WHO I | 20 |
| WHO II | 20 |
| WHO III | 10 |
| NF2 status | |
| Wildtype | 32 |
| Mutation | 18 |

**Supplementary Table 2**.

Oligonucleotides for sgRNA, qRT-PCR, and CUT&Tag-qPCR.

| oligo name | sequence |
|---|---|
| sgRNA | |
| kmt2c_sg_fw | caccgCCTGGACGGCGTAAGAAGCC |
| kmt2c_sg_rev | aaacGGCTTCTTACGCCGTCCAGGc |
| NF2_sg_fw | caccgAAAGATCTACTGCCCTCCTG |
| NF2_sg_rev | aaacCAGGAGGGCAGTAGATCTTTc |
| qRT-PCR primer | |
| KMT2C_fw | TGCGTCTAATGTGCCTTCCTCAG |
| KMT2C_rev | GGTATAAGTCTTGGGTGGGTCTAGC |
| GAPDH_fw | ACACCCACTCCTCCACCTTTG |
| GAPDH_rev | TCCACCACCCTGTTGCTGTAG |
| NF2_fw | GGTTCAGGAGATCACACAACATTTATTC |

| | |
|---|---|
| NF2_rev | GTAAGAAGCCAGGAGCACAGAAG |
| YAP_fw | GTGGATGAGATGGATACAGGTGATAC |
| YAP_rev | GGAATGGCTTCAAGGTAGTCTGG |
| AMOTL2_fw | ACTGGGCGA AAAGCCAGCG |
| *AMOTL2*_rev | TGTGGCAGGTGGCACCTG |
| *CTGF*_fw | ATGGGCCCCGTCCGCGTC |
| *CTGF*_rev | GCAGCATCGGCCGTCGGT |
| *CYR61*_fw | GCTCCCGCATCGCCAGGG |
| *CYR61*_rev | AGCAGGCTGTACAGCTGG |
| *ANKRD1*_fw | CTGCTCTGGAGAATAAACTGCC |
| *ANKRD1*_rev | CAGGCTGTCGATAATTGCTTTG |
| CUT&Tag-qPCR primer | |
| NF2_fw | GTAGTGGTCTGGGCGACCTGAG |
| NF2_rev | TGGCTGTCACCGTCCTCACTC |
| Si-RNA | |
| si-CBP #1_fw | AAAUAAACCUGGACAGUUATT |
| si-CBP #1_rev | UAACUGUCCAGGUUUAUUUTT |
| si-CBP #2_fw | GUGUUAAUGUAGAGAGUAUTT |
| si-CBP #2_rev | AUACUCUCUACAUUAACACTT |
| si-EP300 #1_fw | GGGAGUAAAUGGAGGUGUATT |
| si-EP300 #1_rev | UACACCUCCAUUUACUCCCTT |
| si-EP300 #2_fw | AGGAGGAAGAAGAGAGAAATT |
| si-EP300 #2_rev | UUUCUCUCUUCUUCCUCCUTT |
| Control sequence | UUCUCCGAACGUGUCACGUTT |
| | ACGUGACACGUUCGGAGAATT |

**Supplementary Table 3**: Primers for NF2 Sanger Sequencing

| NF2 exomes | Forward | Reverse |
|---|---|---|
| NF2-E1 | CCGTCTAGGGGTCCCGTC | GCCCGTCTCTAGCTACACC |
| NF2-E2 | AAGCTAGCCATCTCCCCGAG | ACTATACAGCTACAGCGCCC |
| NF2-E3 | TGTCCAAGACTGCTGAGTGAT | CATTACAGAACCAAAGGGGCT |
| NF2-E4 | TGCATAGCCGTCATACCAGT | AGGTTGTACCACATGGTTGAGT |
| NF2-E5 | ATCCCCCTAGTAATGGGCTCC | TGGTGACCCCAAACTAACTCC |
| NF2-E6 | CCTGCCGATTTTACAGCCCT | GACCAACGTTACTCCCAGCTC |
| NF2-E7 | GGTGTCTGCTGTGGCTTTTG | CAGAACCAGGTCCAAGTCCC |
| NF2-E8 | CAGCTGTGACTTCTGTTGGGA | CACCCTCAAAGCCTGGGAATTA |
| NF2-E9 | CTTCCACAGCCCGAGACTTG | AGTATGCGCCAAGTGAGATACC |
| NF2-E10 | GGCCACTAGTAGGGCTTTGG | CTAGCCGGACGGATAGATGC |
| NF2-E11 | CGGGTCAAGACTCAACAGGT | GGTCAGCCCCAAATGTCAGT |
| NF2-E12 | AGTTGGGGAATGTGGCTTGT | GTTCCTGTGCCCAACCAGTA |
| NF2-E13 | CCTCTCCAGCTCCTCTGTCT | CCAAAGCCAAATGGCAGACC |
| NF2-E14 | AGCGAGTGGGAGTTTGTGTT | GTGAGGCTTAGAGCCACCAA |
| NF2-E15 | AACCCTAGATCGCACACCA | GGGCTCAAAATCCACCCTGTA |
| NF2-E16 | TGGGACTGACAGCCAACTTC | GCTTCTCAGGGCTTCAGTGT |

**Supplementary Table 4**. List of agents

| REAGENT or RESOURCE | SOURCE | IDENTIFIER |
|---|---|---|
| Antibodies | | |
| Mouse anti-KMT2C | Thermo Fisher Scientific | #6D1B9 |
| Rabbit anti-NF2 | Santa Cruz Biotechnology | #sc-55575 |
| Rabbit anti-β-actin | Abclonal | #AC026 |
| Rabbit anti-Histone H3 | Abbkine | #ABL1070 |
| Rabbit anti-H3K4me1 | Cell Signaling Technology | #5326 |
| Rabbit anti-H3K4me2 | Cell Signaling Technology | #4658 |
| Rabbit anti-H3K4me3 | Cell Signaling Technology | #9751 |
| Rabbit anti-H3K27me3 | Cell Signaling Technology | #9733 |
| Rabbit anti-H3K27ac | Cell Signaling Technology | #8173 |
| Rabbit anti-EMA | Cell Signaling Technology | #16564 |
| Rabbit anti-MST1 | Proteintech | #22245-1-AP |
| Rabbit anti-P-MST1 | Proteintech | #80093-1-RR |
| Rabbit anti-LATS1 | Proteintech | #27049-1-AP |
| Rabbit anti-P-LATS1 | Abclonal | #AP0904 |
| Rabbit anti-YAP | Cell Signaling Technology | #8418 |
| Rabbit anti-P-YAP | Cell Signaling Technology | #4911 |
| Rabbit anti-ACSL4 | Abcam | #ab155282 |
| Rabbit anti-GPX4 | Abcam | #ab125066 |
| Rabbit anti-CBP | GeneTex | #GTX101249 |
| Rabbit anti-EP300 | Abclonal | #A13016 |
| Rabbit anti-KI67 | Proteintech | #27309-1-AP |
| ABflo® 594-conjugated Goat anti-Mouse IgG (H+L) | Abclonal | #AS054 |
| ABflo® 488-conjugated Goat anti-Rabbit IgG (H+L) | Abclonal | #AS073 |

| | | |
|---|---|---|
| Chemicals | | |
| Verteporfin | Bioss | #bioss-D50658 |
| Trichostatin A | Beyotime | #ST1713 |
| Erastin | Selleck Chemicals | #S7242 |
| RSL3 | Selleck Chemicals | #S8155 |
| Ferrostatin-1 | Selleck Chemicals | #S7243 |
| Critical Commercial kits | | |
| RNA Extraction Kit | Beyotime | #ROO24 |
| Nuclear and Cytoplasmic Protein Extraction Kit | Beyotime | #P0027 |
| Hoechst 33342 | Beyotime | #C1025 |
| TUNEL Apoptosis Detection Kit | Beyotime | #C1088 |
| ROS assay kit | Beyotime | #SOO34S |
| GSH Assay Kit | Beyotime | #S0053 |
| FerroOrange probe | Dojindo | #F374 |
| MDA detection kit | Dojindo | #M496 |
| BODIPY-C11 dye (Thermo Fisher, #D3861 | | |
| The Hyperactive Universal CUT&Tag Assay Kit for Illumina Pro | Vazyme | #TD904 |

# Supplementary Figures

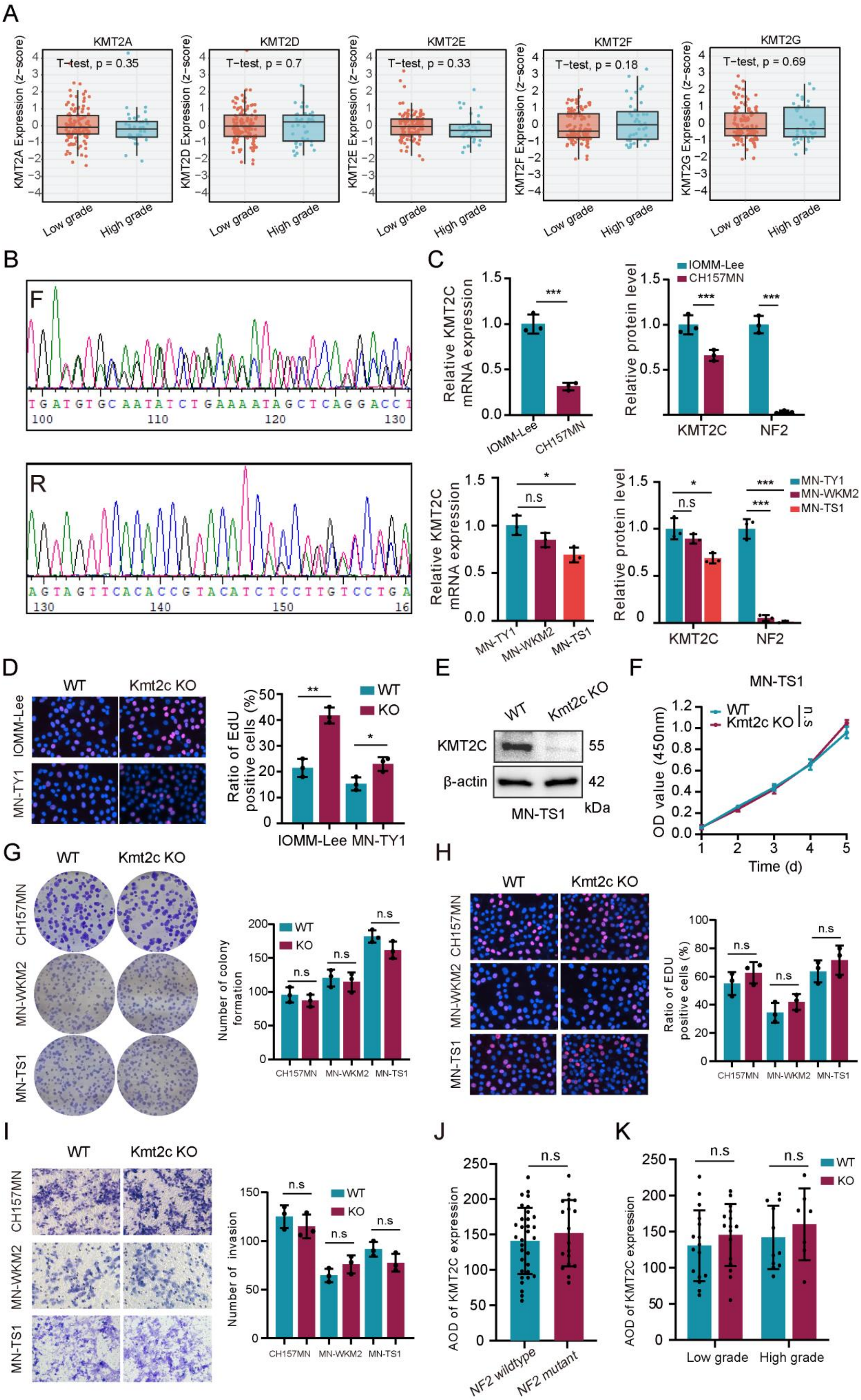

**Figure S1. (A)** Relative mRNA expression of KMT2 family in meningioma samples according to GEO dataset GSE136661. Data are presented as means ± SEM, normalized as Z-scores and analyzed by unpaired two-tailed Student's t-test. **(B)** Representative Sanger sequencing chromatograms showing NF2 mutation status in MN-WKM2 cells. **(C)** Relative KMT2C mRNA and KMT2C protein expression levels in meningioma cell lines and primary meningioma cells in meningioma cell lines and primary meningioma cells. **(D)** EdU assay and quantitative analysis in WT and KMT2C-KO IOMM-Lee and MN-TY1 cells. (n = 3). **(E)** Western blot analysis of KMT2C expression in transfected primary meningioma cells. **(F)** Cell viability determined by CCK-8 assays in KMT2C-WT and KMT2C-KO MN-TS1 cells (n = 3). **(G)** Representative images and quantification of colony formation in KMT2C-WT and KMT2C-KO CH157MN, MN-WKM2 and MN-TS1 cells (n = 3). **(H)** EdU assay and quantitative analysis in WT and KMT2C-KO CH157MN, MN-WKM2 and MN-TS1 cells. (n = 3). **(I)** Representative transwell invasion assay images and quantification of invaded KMT2C-WT and KMT2C-KO CH157MN, MN-WKM2 and MN-TS1 cells after 24 h (n = 3). **(J)** Quantitative immunohistochemical analysis of KMT2C expression in meningioma samples with different NF2 statuses. **(K)** Quantitative analysis of KMT2C expression stratified by both NF2 status and WHO tumor grade, showing no significant difference in KMT2C levels across NF2 statuses within each grade. Data are presented as means ± SD and analyzed using two-way ANOVA with post hoc test or unpaired two-tailed Student's *t*-test. n.s., $P > 0.05$; *P < 0.05, **P < 0.01, ***P < 0.001.

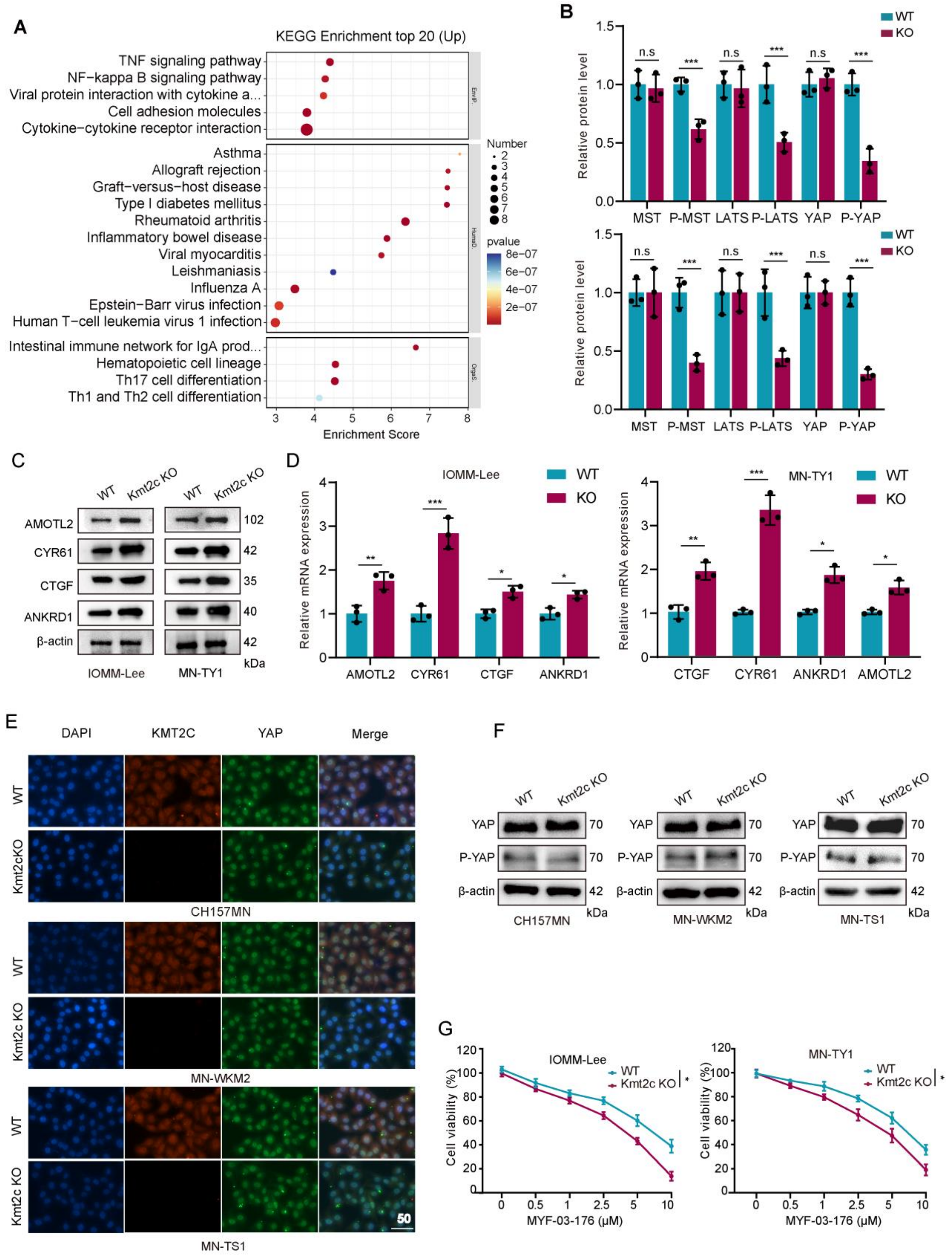


**Figure S2. (A)** KEGG pathway analysis of upregulated targets in Kmt2c KO transcriptome. **(B)** Quantification of Western blot analysis in WT and KMT2C-KO IOMM-Lee and MN-TY1 cells. **(C)** Western blot analysis of indicated proteins in WT and KMT2C-KO IOMM-Lee and MN-TY1 cells. **(D)** Relative mRNA expression levels in WT and KMT2C-KO IOMM-Lee and MN-TY1 cells. **(E)** Immunofluorescence staining of YAP in WT and KMT2C-KO CH157MN,

MN-WKM2 and MN-TS1 cells. Scale bar, 50 μm. **(F)** Western blot analysis in WT and KMT2C-KO CH157MN, MN-WKM2 and MN-TS1 cells. **(G)** WT and KMT2C-KO IOMM-Lee and MN-TY1 cells were exposed to the respective indicated dose of MYF-03-176 (0–10 μM) for 24 h and the cell viability was examined using CCK8 assays. ($n = 3$). **(C, F)** Representative western blots are shown from $n = 3$ independent biological replicates. Data are presented as means ± SD and analyzed by two-way ANOVA with post hoc test and one-way ANOVA. n.s for $P > 0.05$, $^{*}P < 0.05$, $^{**}P < 0.01$, $^{***}P < 0.001$.

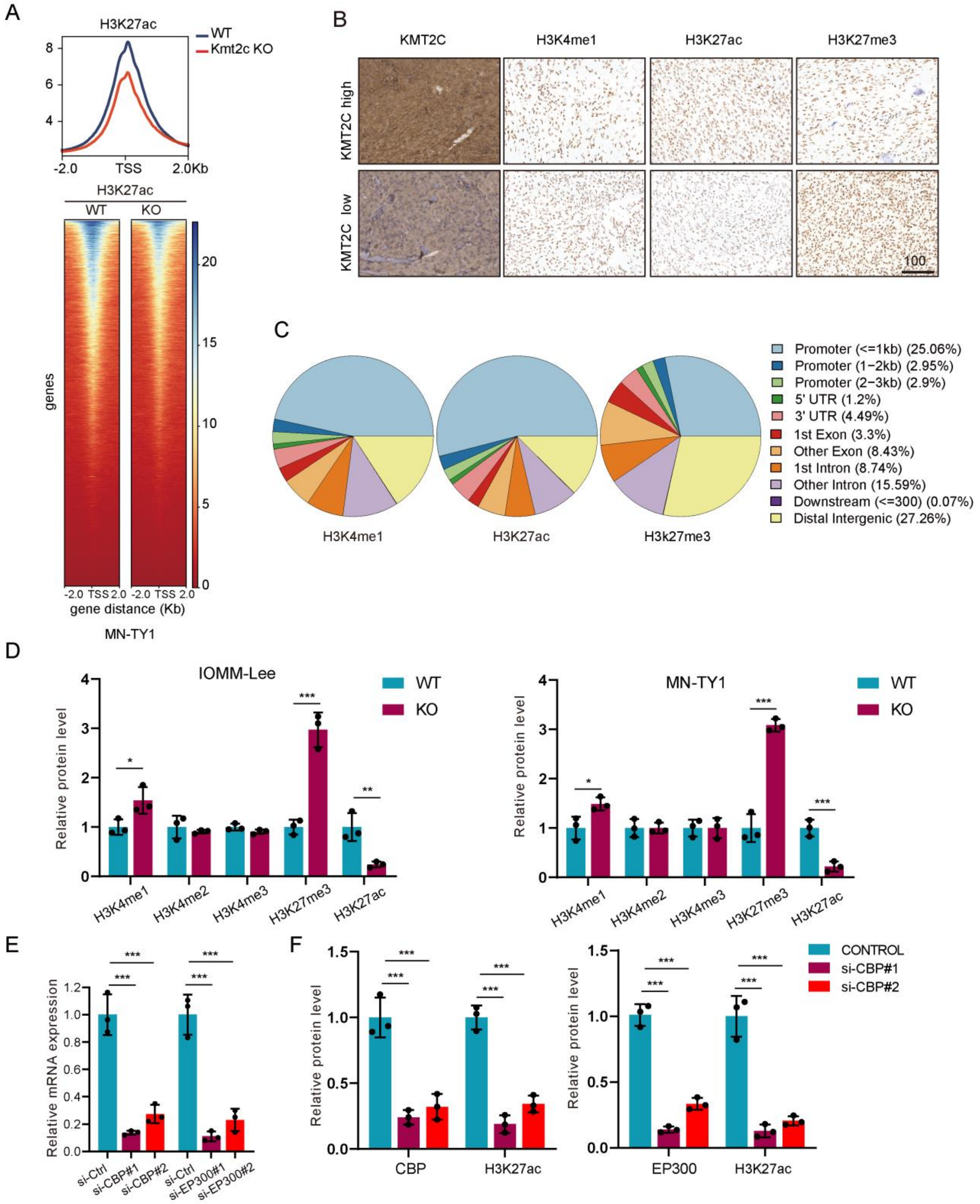


**Figure S3. (A)** Average signaling intensity curves (up) and heatmap (down) of CUT&Tag-seq reads for H3K27ac in WT and KMT2C-KO MN-TY1 cells. **(B)** Representative immunohistochemical staining of H3K4me1, H3K27ac, and H3K27me3 in meningioma samples with different KMT2C expression levels. **(C)** Percentage of H3K4me1、H3K27me3 and H3K27ac bound peaks at each chromatin state. **(D)** Quantification of histone modification levels in KMT2C-WT and KMT2C-KO cells. **(E)** Relative CBP and EP300 mRNA expression levels. **(F)**

Quantification of protein levels in IOMM-Lee cells after 72 h of CBP or EP300 knockdown. Data are presented as means ± SD and analyzed by two-way ANOVA with post hoc test and one-way ANOVA. n.s for $P > 0.05$, *$P < 0.05$, **$P < 0.01$, ***$P < 0.001$.

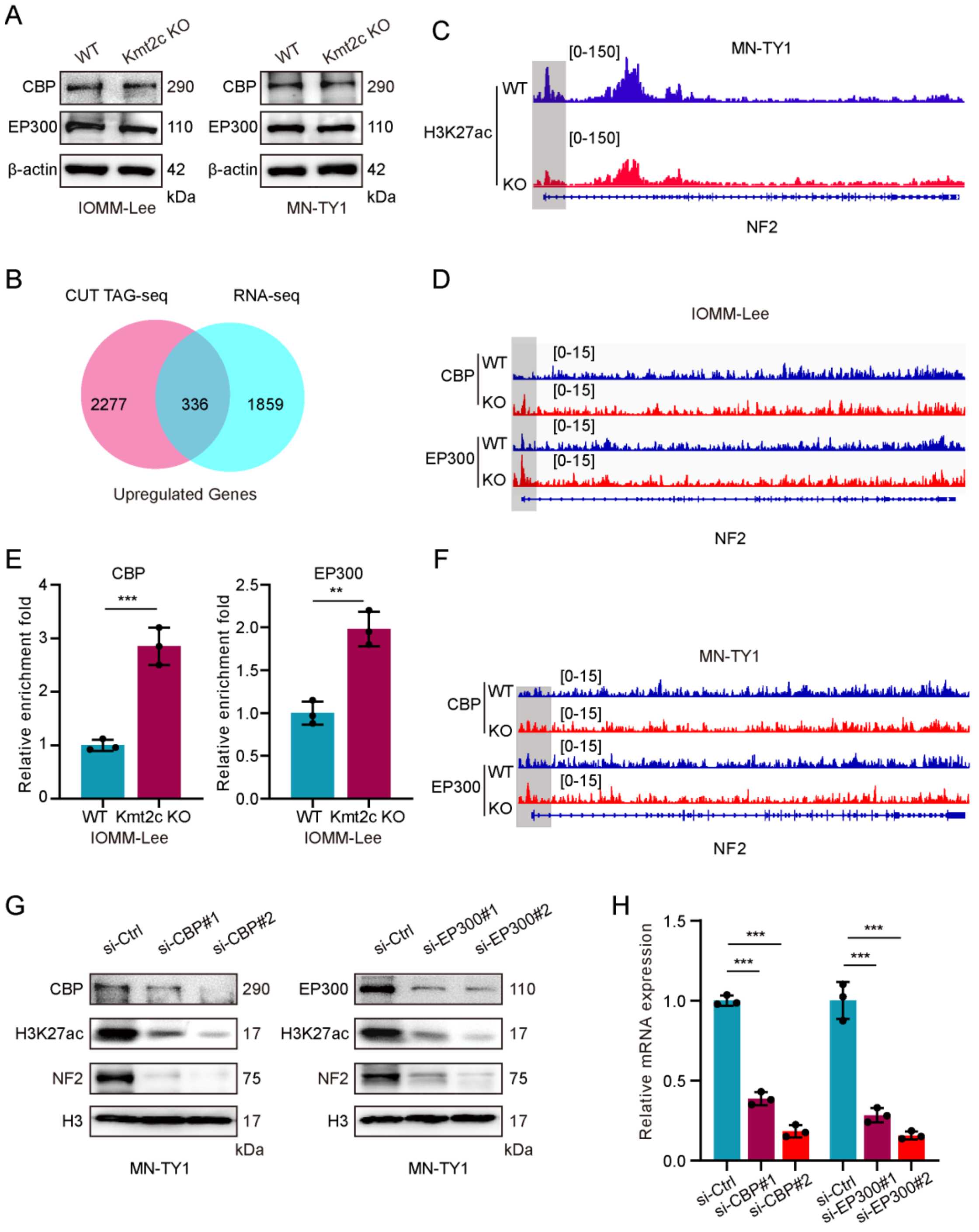


**Figure S4. (A)** Western blot analyses of CBP and EP300 protein levels in WT and KMT2C-KO IOMM-Lee and MN-TY1 cells. **(B)** Venn diagram of overlap genes between H3K4me1-bound genes from CUT&Tag-seq data and upregulated genes by KMT2C loss from RNA-seq data. **(C)** Genome tracks of H3K27ac peaks in WT, KMT2C-KO MN-TY1 cells at the NF2 gene locus. **(D, F)** Genome browser tracks illustrating CBP and EP300 occupancy at the *NF2* gene locus in WT and KMT2C-KO cells. **(E)** CUT&Tag-qPCR analysis quantifying CBP and EP300 binding at the *NF2*

promoter in WT and KMT2C-KO IOMM-Lee cells ($n = 3$). **(G)** Western blotting of H3K27ac and NF2 in MN-TY1 cells transfected with CBP or EP300 siRNA (72 h). **(H)** Relative CBP and EP300 mRNA expression levels in MN-TY1 cells. **(A, G)** Representative western blots are shown from $n = 3$ independent biological replicates. Data are presented as means ± SD and analyzed by unpaired two-tailed Student’s t-test. n.s. for $P > 0.05$, $^{*}P < 0.05$, $^{**}P < 0.01$, $^{***}P < 0.001$.

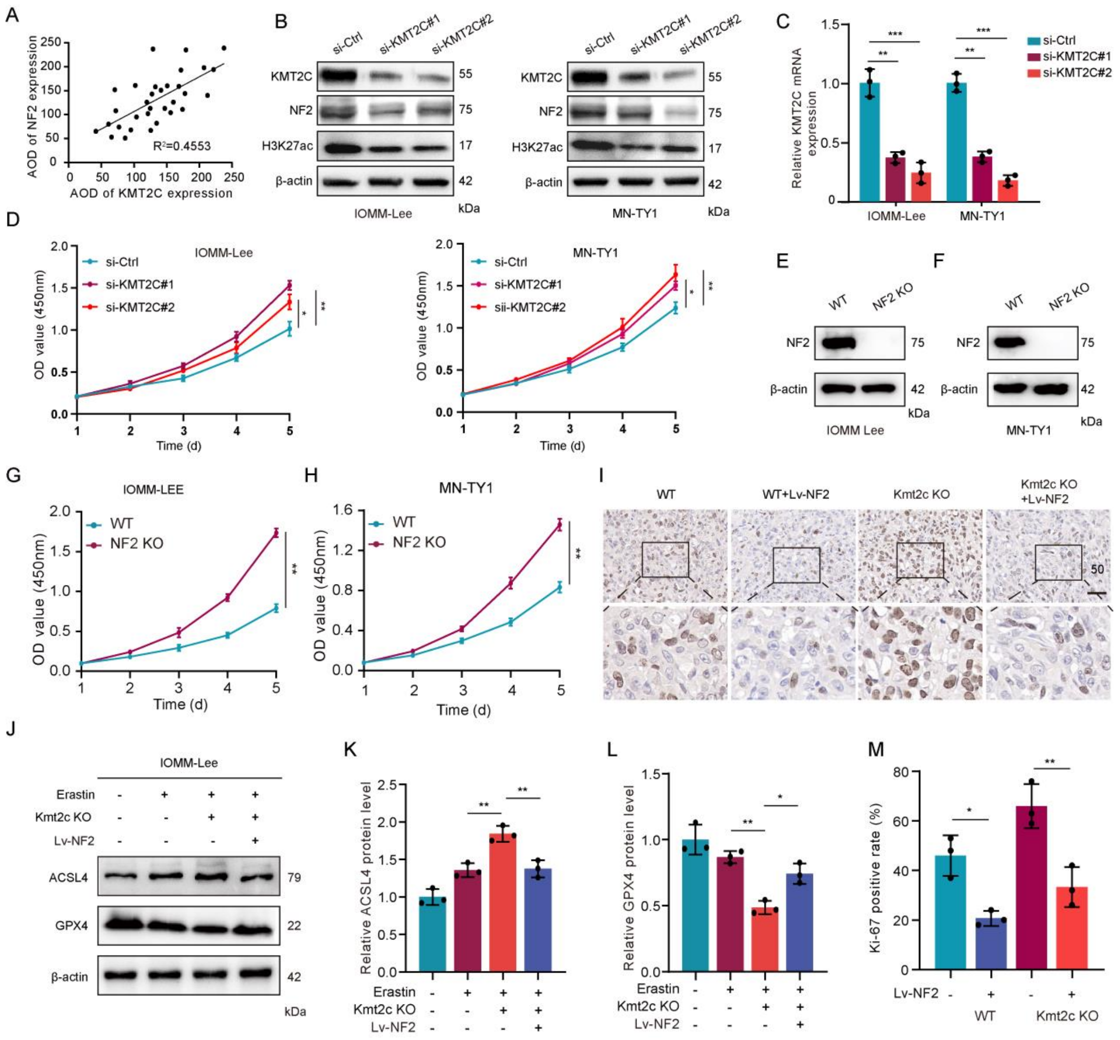


**Figure S5. KMT2C regulates NF2 expression and modulates tumor cell proliferation.** **(A)** Correlation between KMT2C and NF2 protein expression across clinical samples (r² = 0.4553). (n = 32). **(B)** Western blot analysis of KMT2C, NF2 and H3K27ac in WT and KMT2C-knockdown IOMM-Lee and MN-TY1 cells. **(C)** Relative mRNA expression of KMT2C in WT and KMT2C-knockdown IOMM-Lee and MN-TY1 cells. **(D)** CCK-8 assays showing cell proliferation in WT and KMT2C-knockdown IOMM-Lee and MN-TY1 cells. **(E-F)** Western blotting of NF2 in WT and NF2 KO IOMM-Lee and MN-TY1 cells. **(G-H)** CCK-8 assays in indicated WT and NF2-KO IOMM-Lee and MN-TY1 cells. **(I, M)** IHC assay for Ki-67. Scale bar is 50 μm. (n = 3). **(J-L)** WT and KMT2C-KO IOMM-Lee cells transfected with NF2 overexpression plasmid were stimulated with 6 μM Erastin for 24 hours to trigger ferroptosis. Western blotting assays and densitometry quantification for ACSL4, and GPX4. (n = 3). **(B, E, F, J)** Representative western

blots are shown from n = 3 independent biological replicates. Data are presented as means ± SD and analyzed by two-way ANOVA with post hoc test and unpaired two-tailed Student's t-test. n.s for $P > 0.05$, *$P < 0.05$, **$P < 0.01$, ***$P < 0.001$.

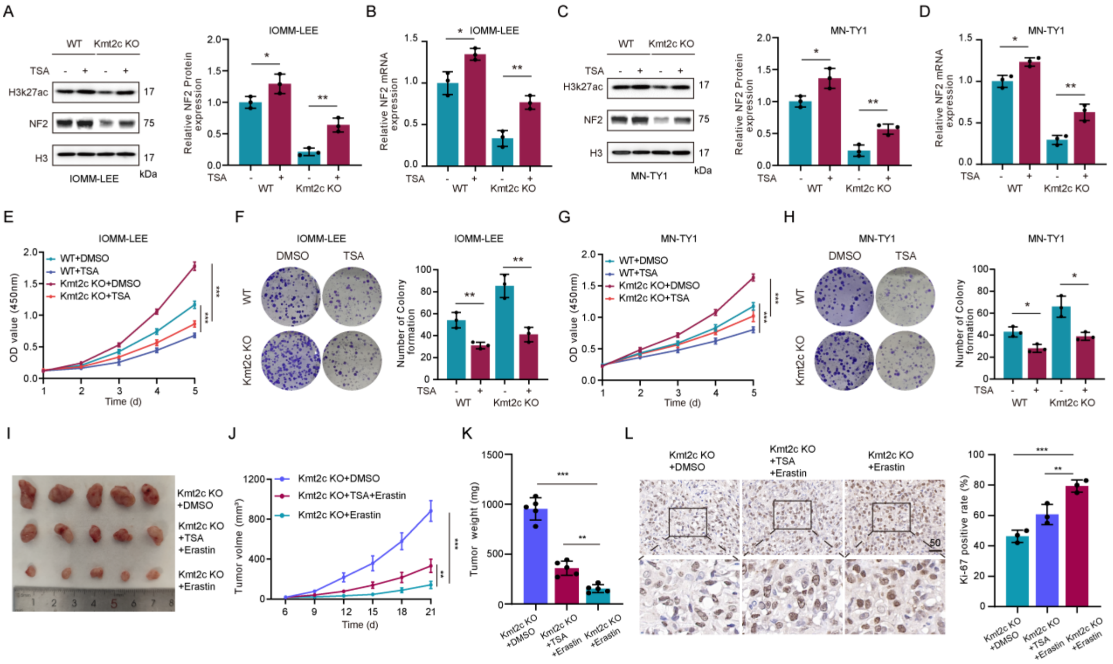


**Figure S6. TSA treatment partially reverses Erastin-induced ferroptosis sensitivity in KMT2C-deficient meningiomas. (A, C)** Western blotting analysis of NF2 protein levels in KMT2C-WT and KMT2C-KO IOMM-Lee **(A)** and MN-TY1 **(C)** cells treated with 100 μM trichostatin A (TSA) for 24 h. (n=3). **(B, D)** qRT-PCR analysis of NF2 mRNA expression in KMT2C-WT and KMT2C-KO IOMM-Lee **(B)** and MN-TY1 **(D)** cells treated with 100 μM trichostatin A (TSA) for 24 h. (n=3). **(E, G)** CCK-8 assays in indicated WT and KMT2C-KO IOMM-Lee and MN-TY1 cells treated with DMSO or 100 μM TSA. (n=3). **(F, H)** Colony formation assay and quantitative analysis in WT and KMT2C-KO IOMM-Lee and MN-TY1 cells treated with DMSO or 100 μM TSA. (n=3). **(I)** Representative images of subcutaneous tumors derived from KMT2C-WT and KMT2C-KO IOMM-Lee xenografts treated with vehicle, Erastin, TSA, or Erastin + TSA. **(J)** Tumor growth curves showing tumor volumes measured over time in each treatment group (n = 5). **(K)** Tumor weights at the endpoint of treatment (n = 5). **(L)** Representative immunohistochemical staining of Ki-67 and quantitative analysis of Ki-67–positive tumor cells in each group. Scale bar, 50 μm. **(A, C)** Representative western blots are shown from n = 3 independent biological replicates. Data are presented as means ± SD and analyzed by two-way ANOVA with post hoc test and unpaired two-tailed Student's t-test. n.s for $P > 0.05$, *$P < 0.05$, **$P < 0.01$, ***$P < 0.001$.
