# Supplementary material for: KMT2C Loss Promotes NF2‐Wildtype Meningioma Progression and Ferroptosis Sensitivity via Epigenetic Repression of Hippo Signaling
Source: Adv Sci (Weinh). 2026 Feb 5;13(20):e22756. doi: 10.1002/advs.202522756 (PMC13067829; doi:10.1002/advs.202522756)

Raw Data of Blots

Figure. 1. C

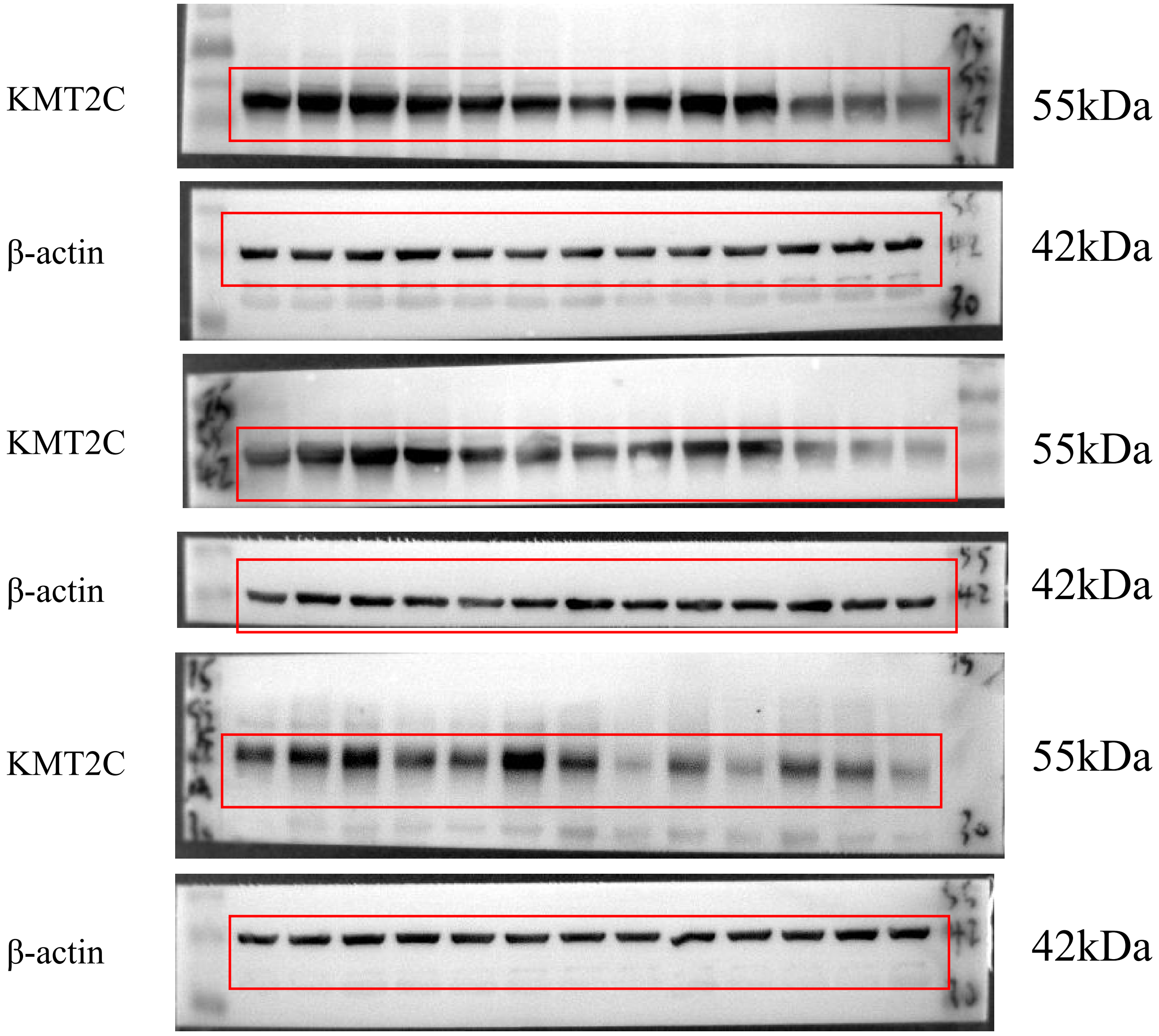
KMT2C
55kDa
β-actin
42kDa
KMT2C
55kDa
β-actin
42kDa
KMT2C
55kDa
β-actin
42kDa

Figure. 1. G

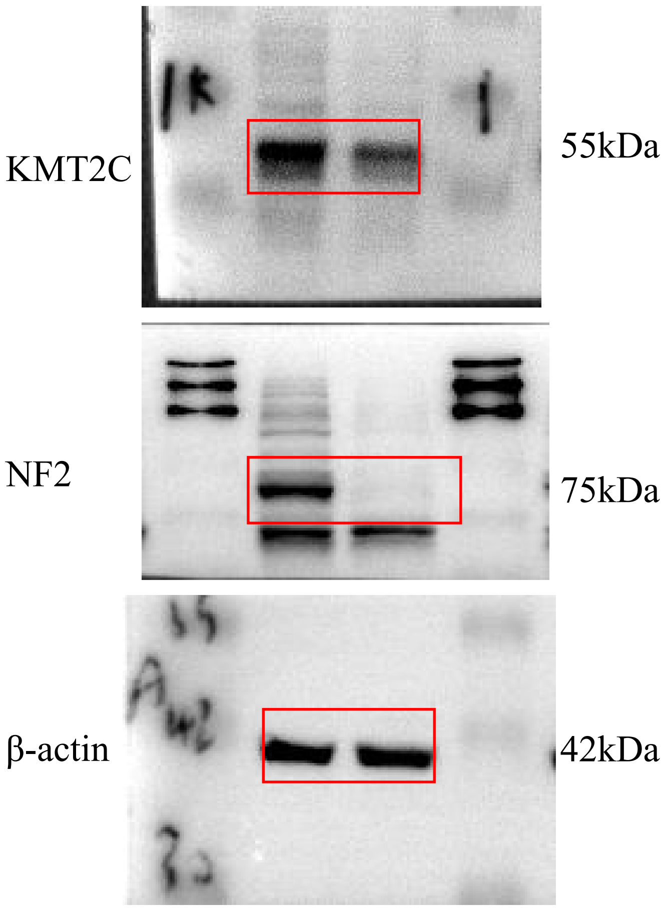

Figure. 1. I

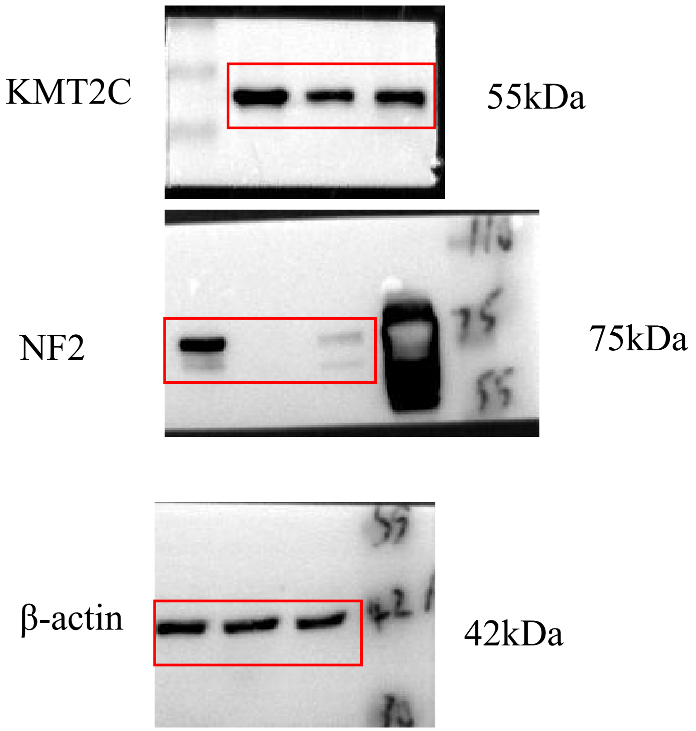

Figure. 2. A

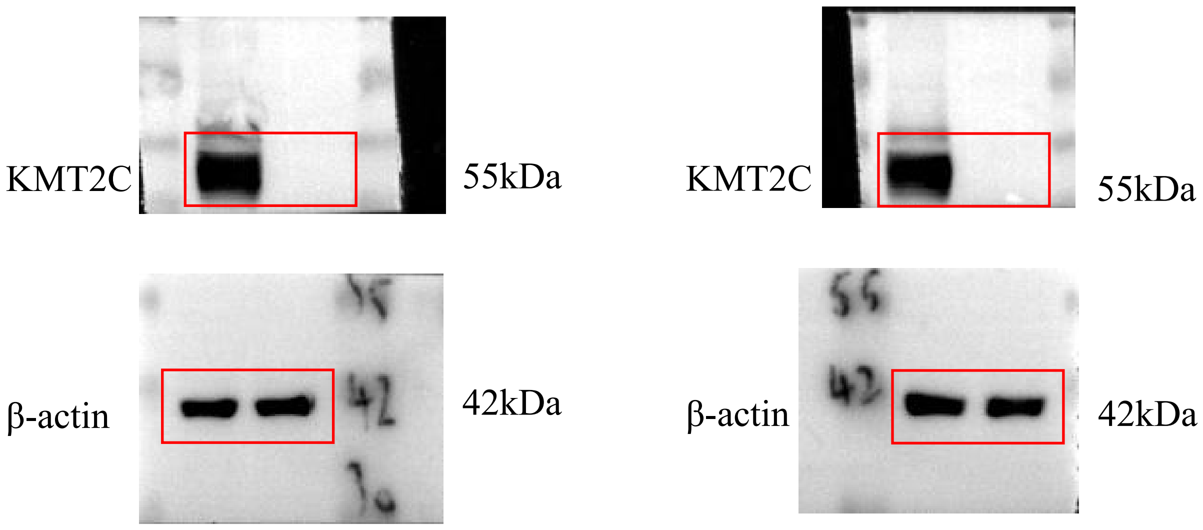

Figure. 2. A

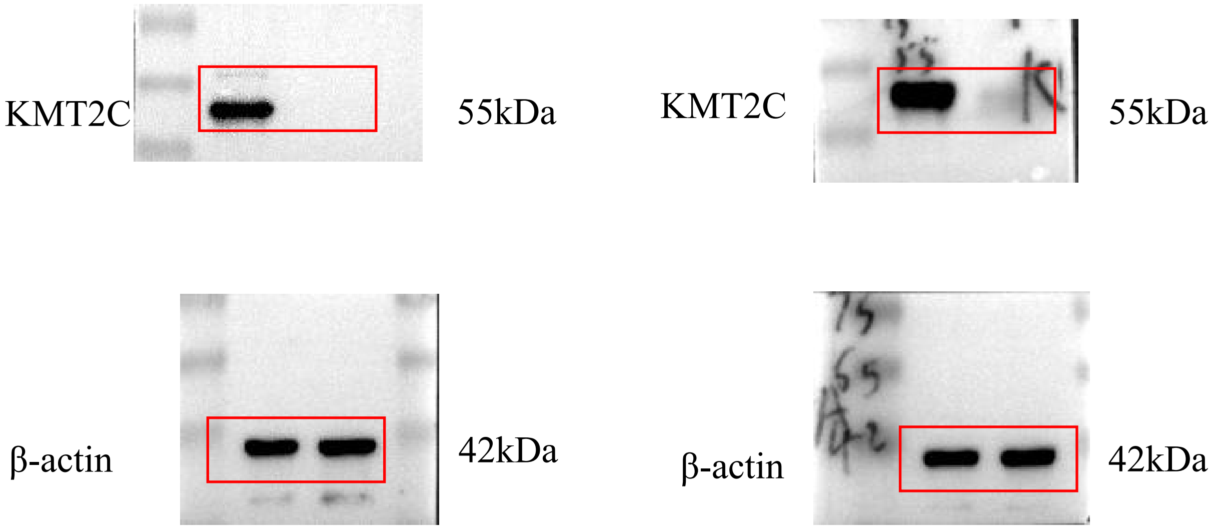

Figure. 3. C

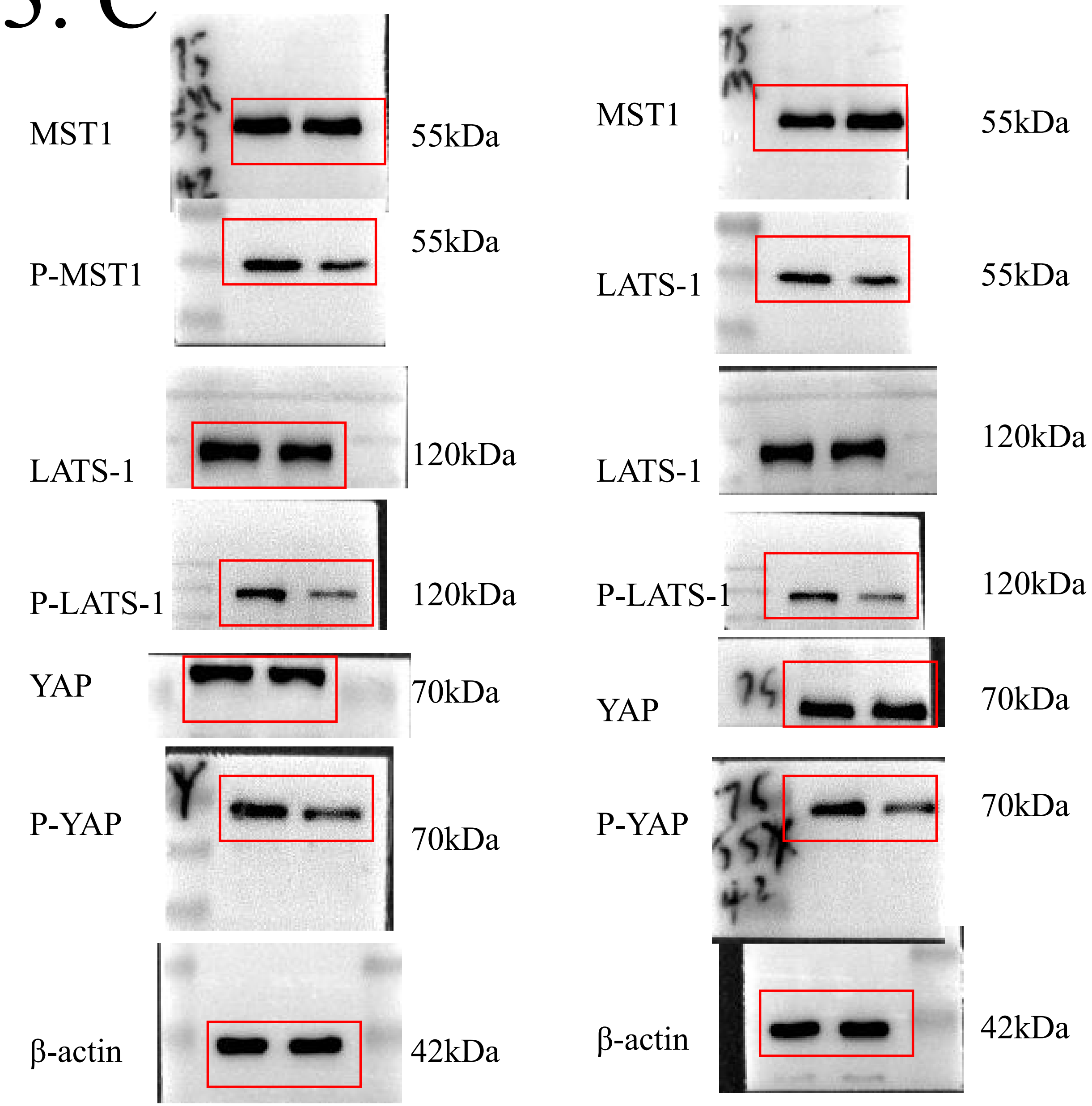
MST1
55kDa
P-MST1
55kDa
LATS-1
120kDa
P-LATS-1
120kDa
YAP
70kDa
P-YAP
70kDa
β-actin
42kDa
MST1
55kDa
LATS-1
55kDa
LATS-1
120kDa
P-LATS-1
120kDa
YAP
70kDa
P-YAP
70kDa
β-actin
42kDa

Figure. 3. E

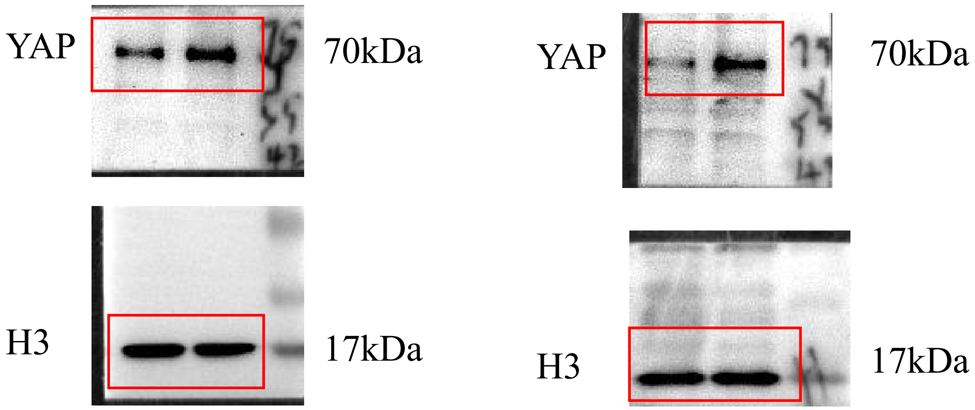

Figure. 4. H

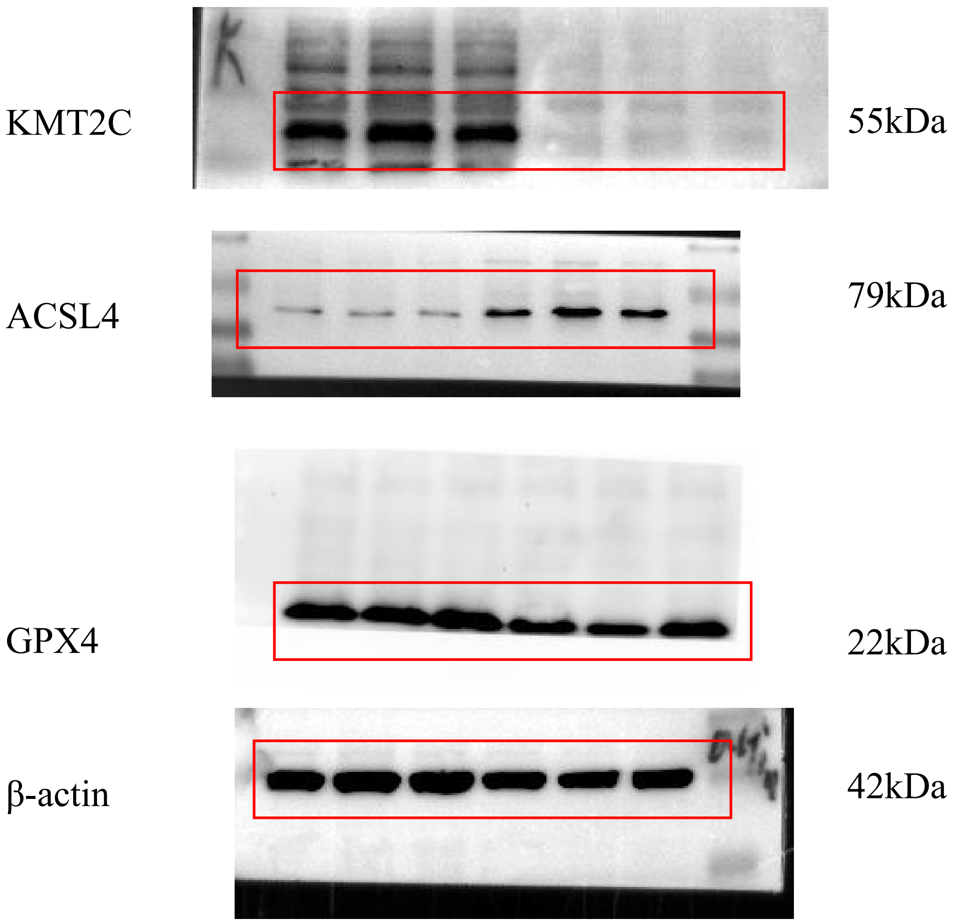

Figure. 4. Q

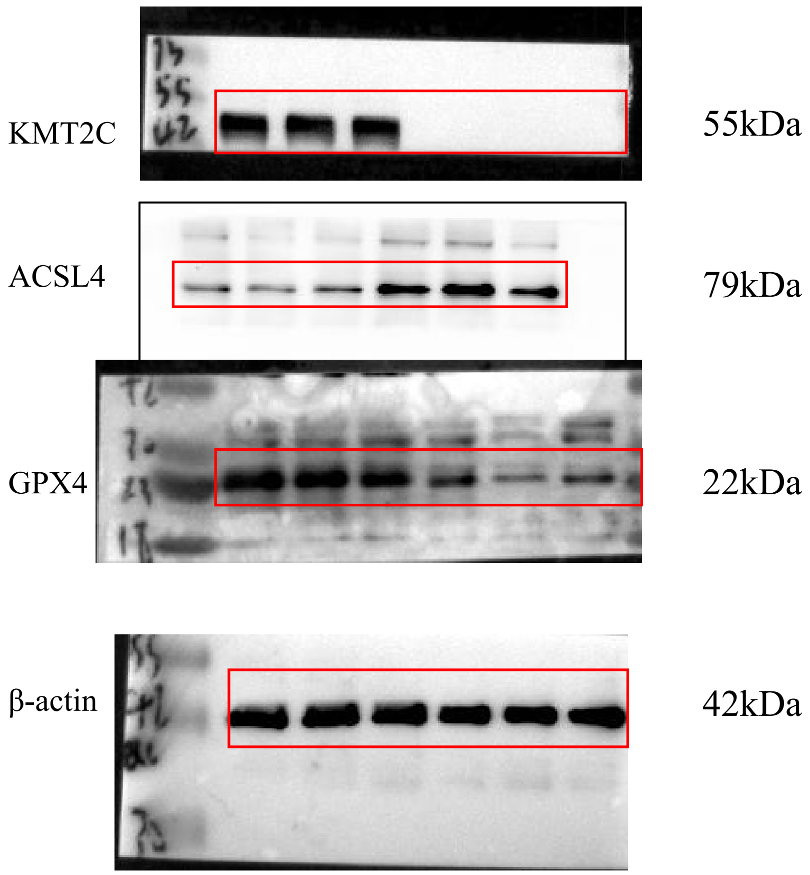

Figure. 5. C

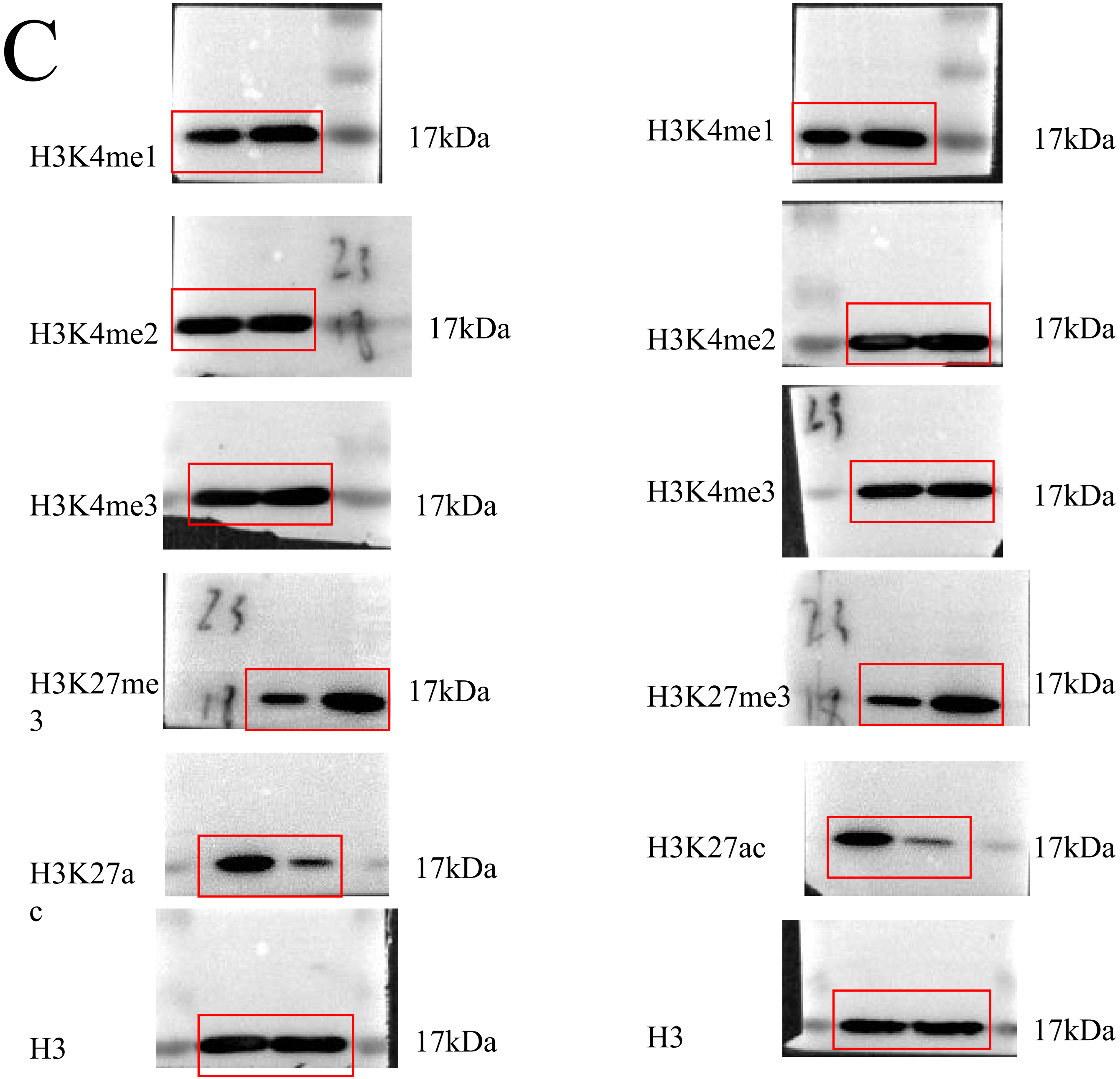
H3K4me1
17kDa
H3K4me2
17kDa
H3K4me3
17kDa
H3K27me
3
17kDa
H3K27a
c
17kDa
H3
17kDa
H3K4me1
17kDa
H3K4me2
17kDa
H3K4me3
17kDa
H3K27me3
17kDa
H3K27ac
17kDa
H3
17kDa

Figure. 5. E

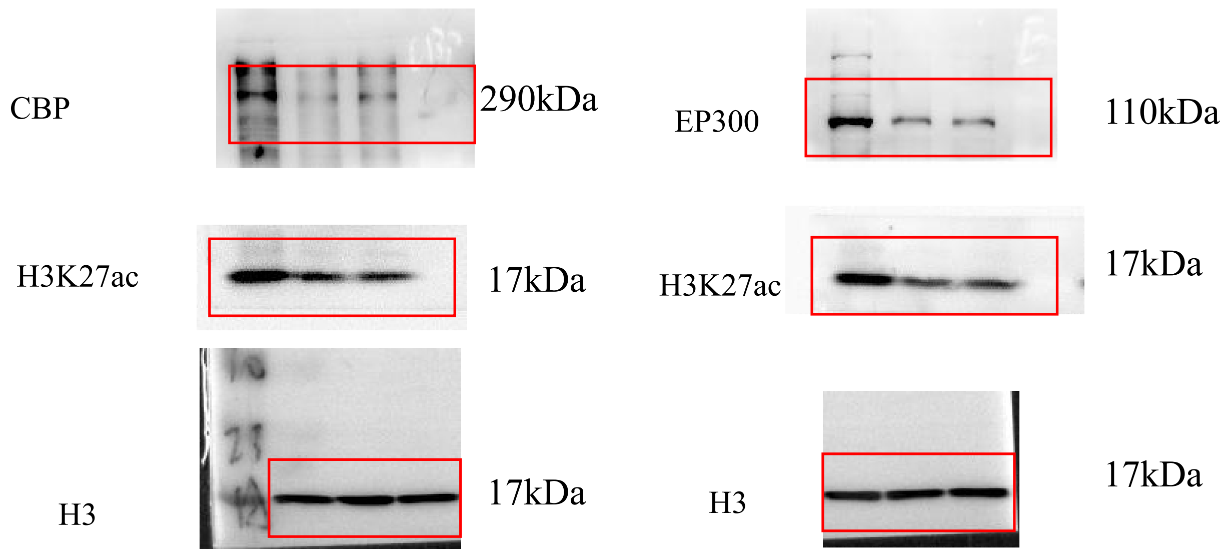

CBP
290kDa
EP300
110kDa
H3K27ac
17kDa
H3K27ac
17kDa
H3
17kDa
H3
17kDa

Figure. 7. D

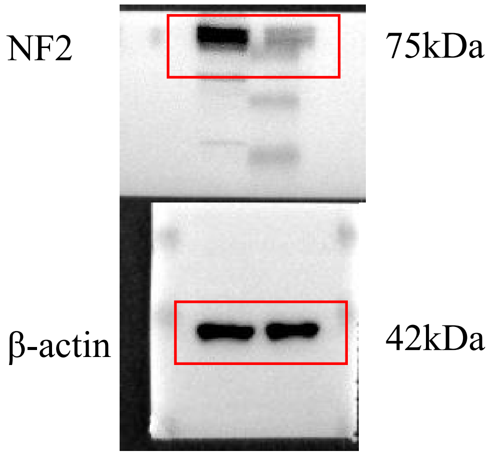

Figure. 7. D

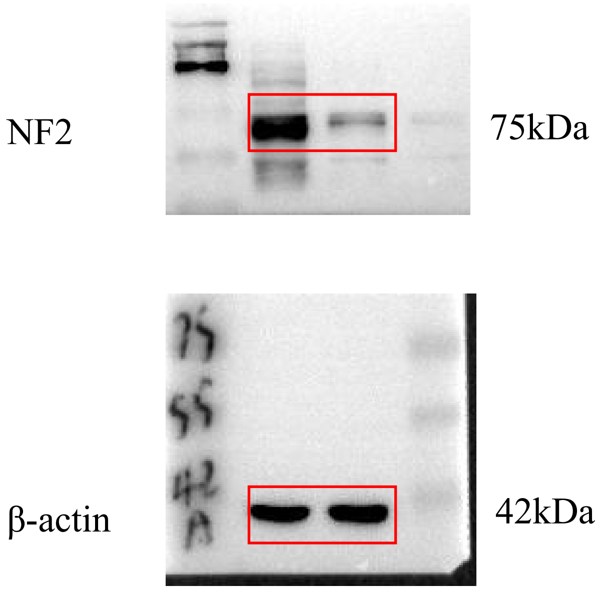

Figure. 7. F

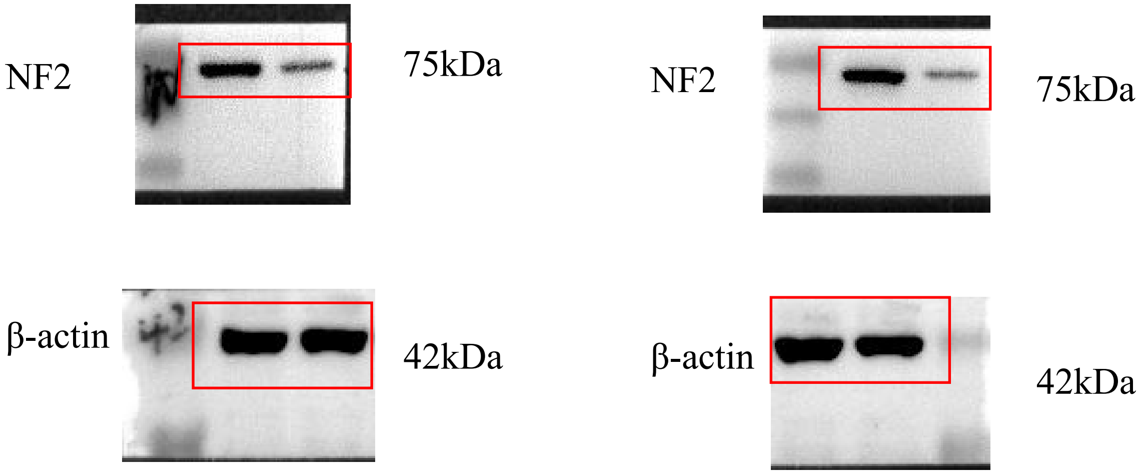

Figure. 7. H

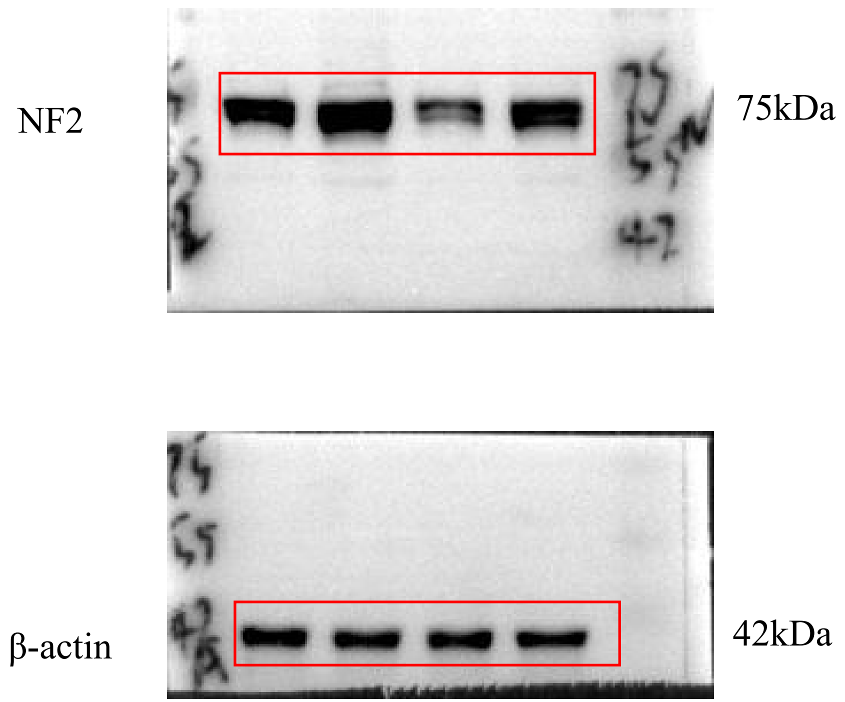

Figure. S1. E

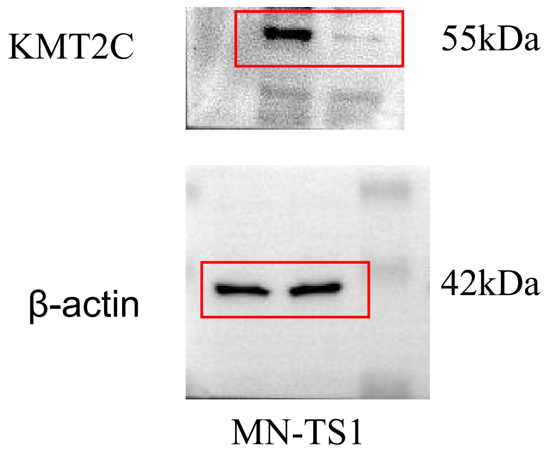

Figure. S2. B

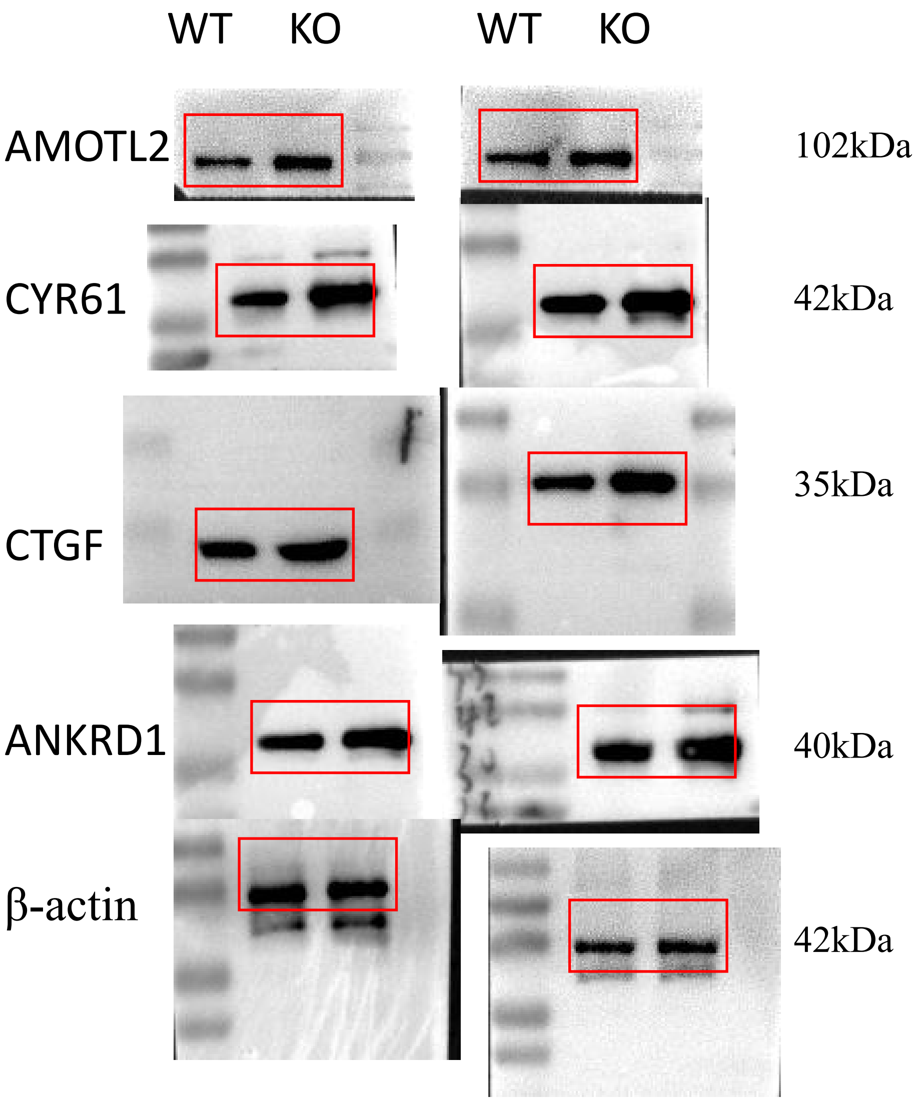

WT
KO
WT
KO
AMOTL2
102kDa
CYR61
42kDa
CTGF
35kDa
ANKRD1
40kDa
β-actin
42kDa

Figure. S2. F

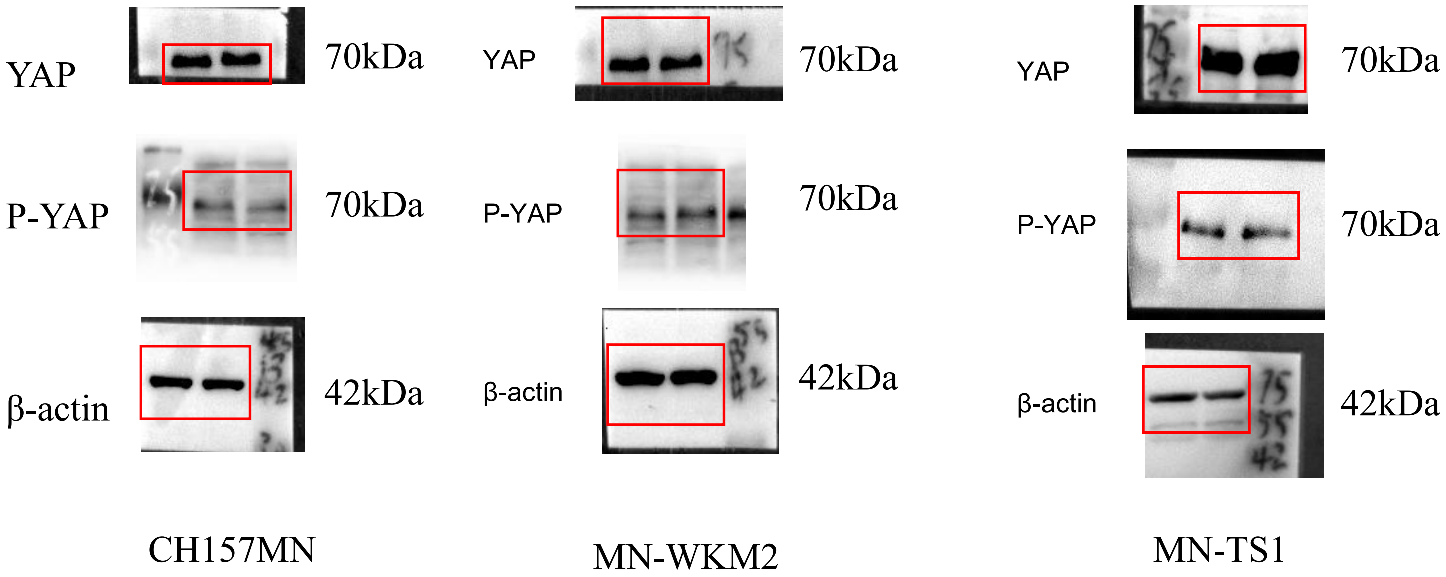
YAP
70kDa
P-YAP
70kDa
β-actin
42kDa
CH157MN
YAP
70kDa
P-YAP
70kDa
β-actin
42kDa
MN-WKM2
YAP
70kDa
P-YAP
70kDa
β-actin
42kDa
MN-TS1

Figure. S4. A

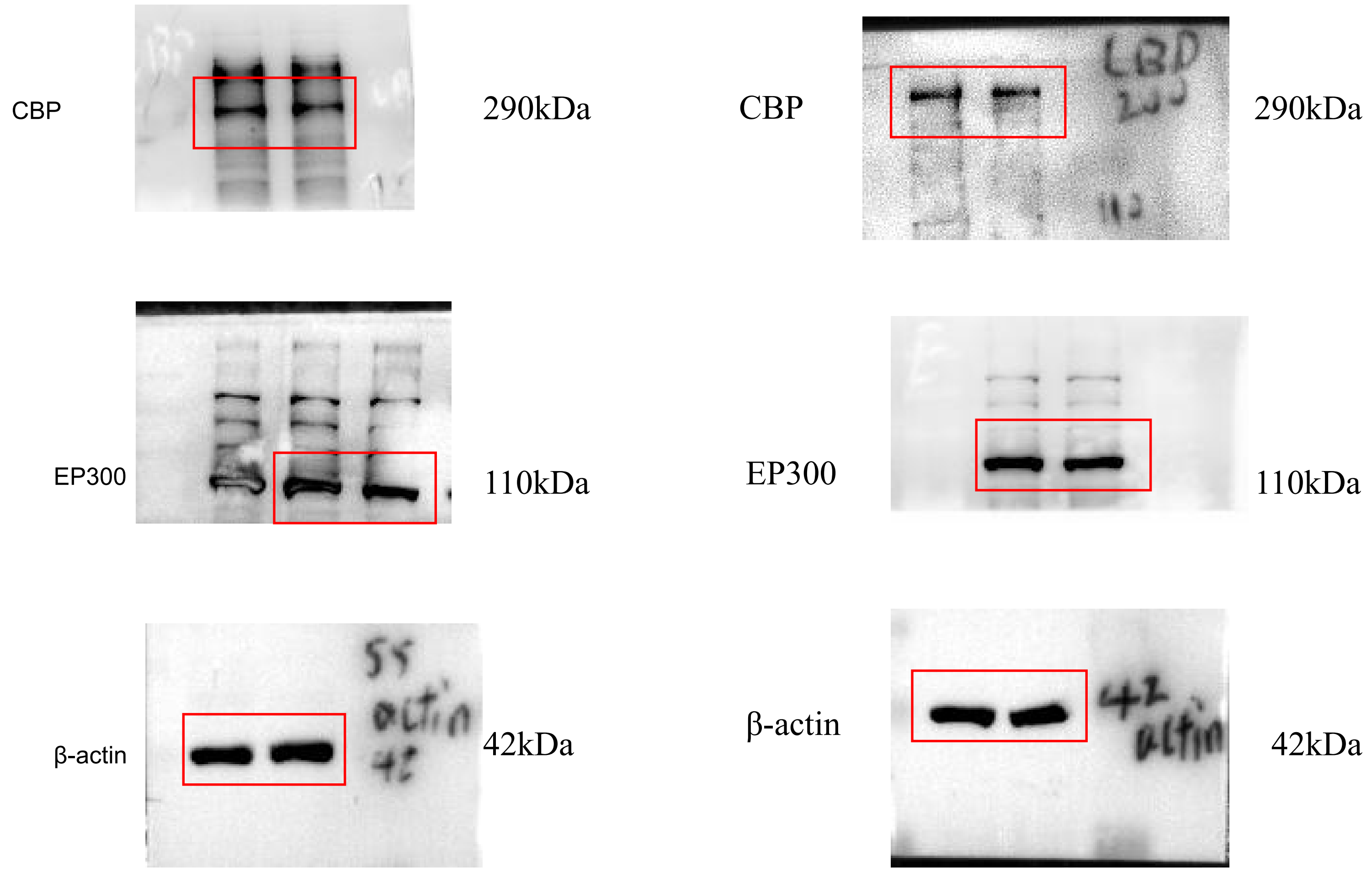
CBP
290kDa
CBP
290kDa
EP300
110kDa
EP300
110kDa
β-actin
42kDa
β-actin
42kDa

Figure. S4. G

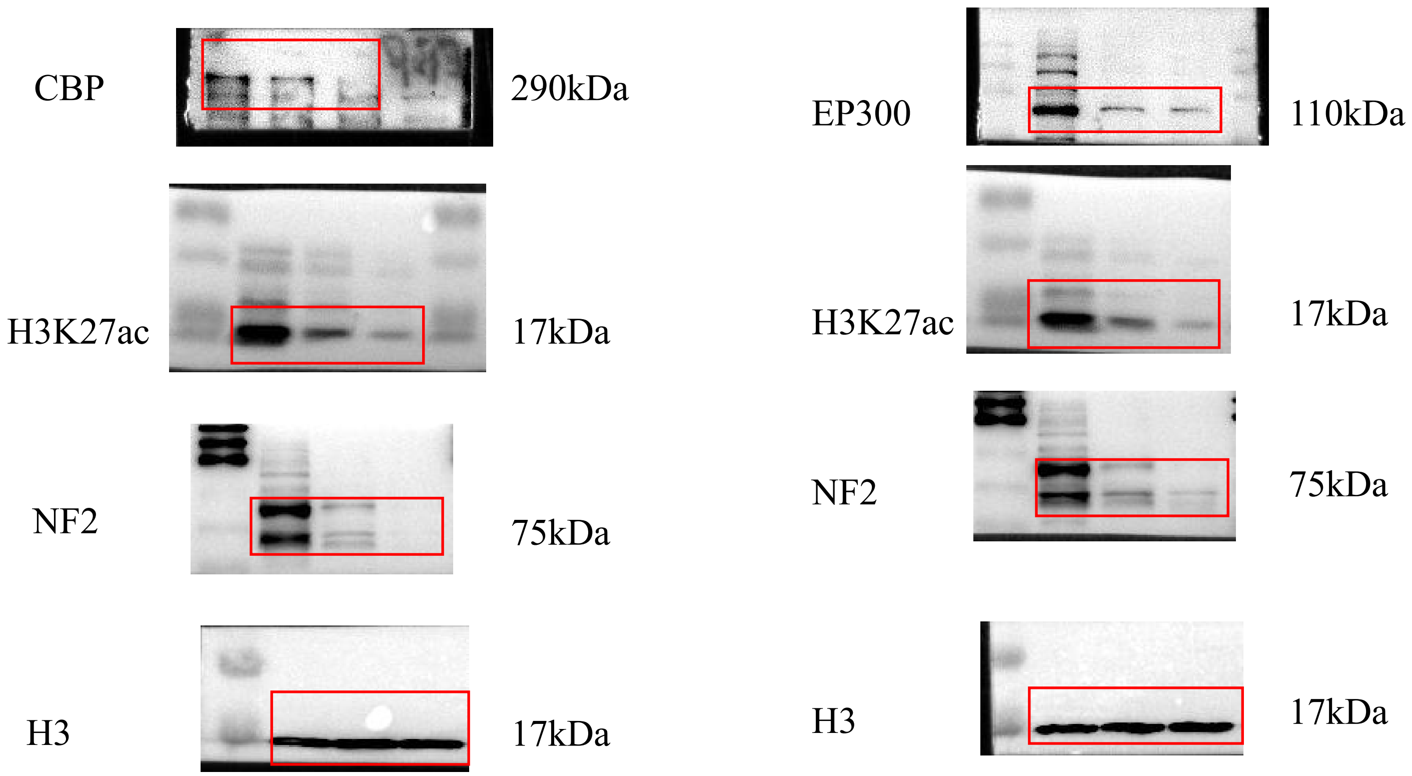

CBP
290kDa
EP300
110kDa
H3K27ac
17kDa
H3K27ac
17kDa
NF2
75kDa
NF2
75kDa
H3
17kDa
H3
17kDa

Figure. S5. B

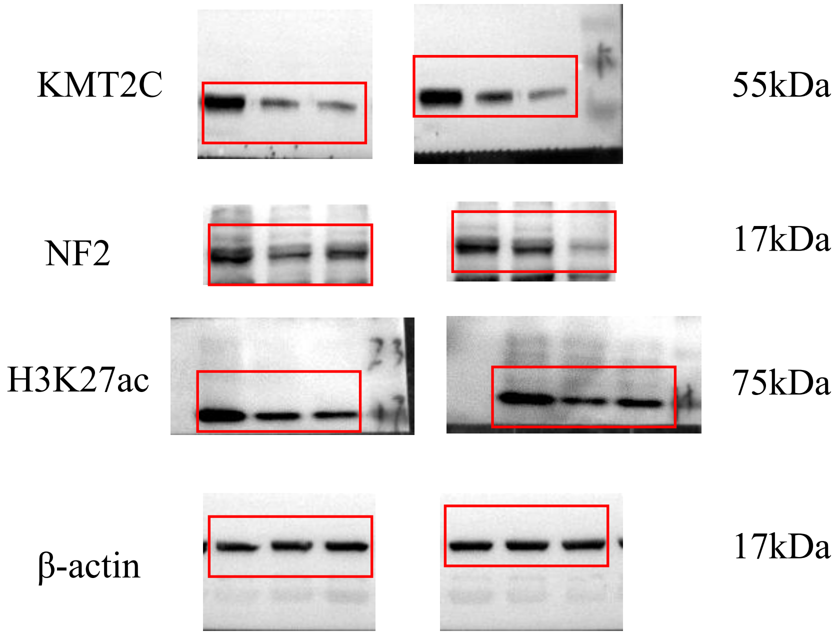

Figures. S5. E-F

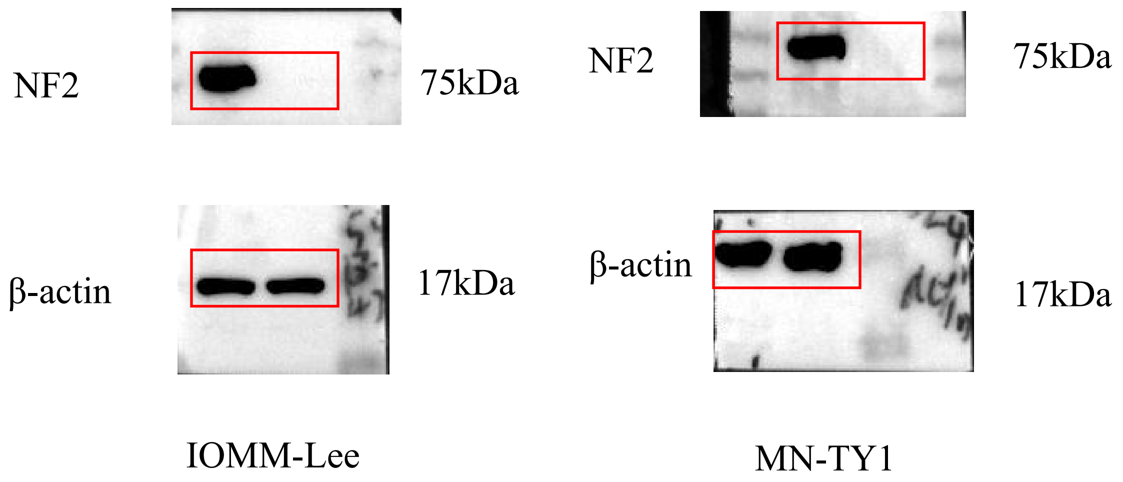

Figure. S5. J

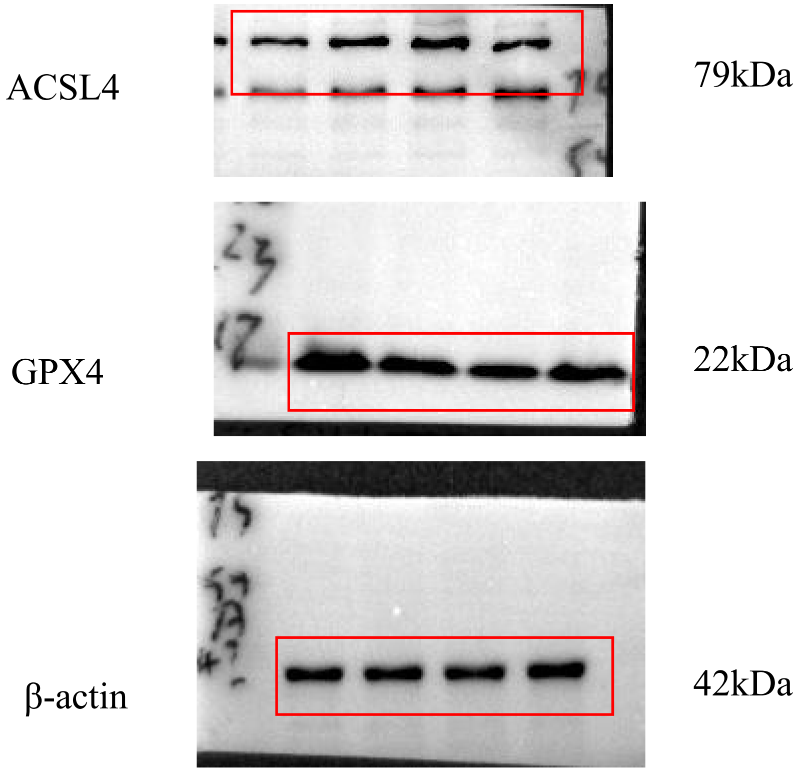

Figure. S6. A

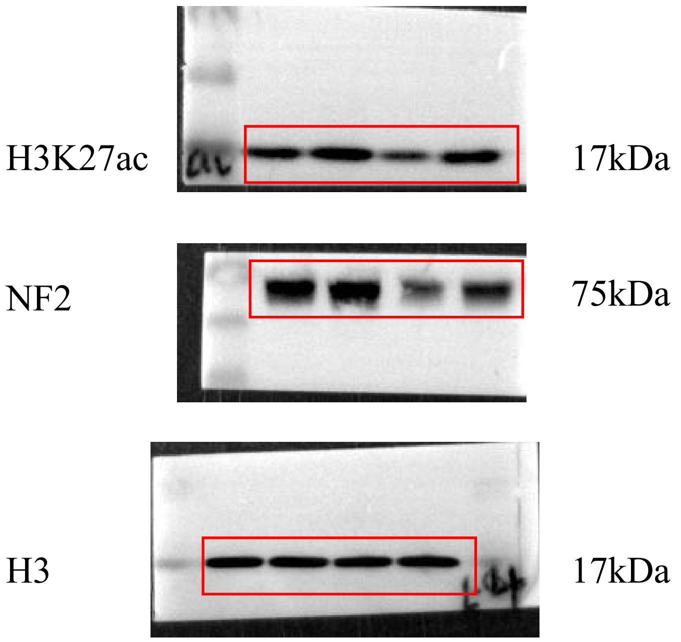

Figure. S6. C

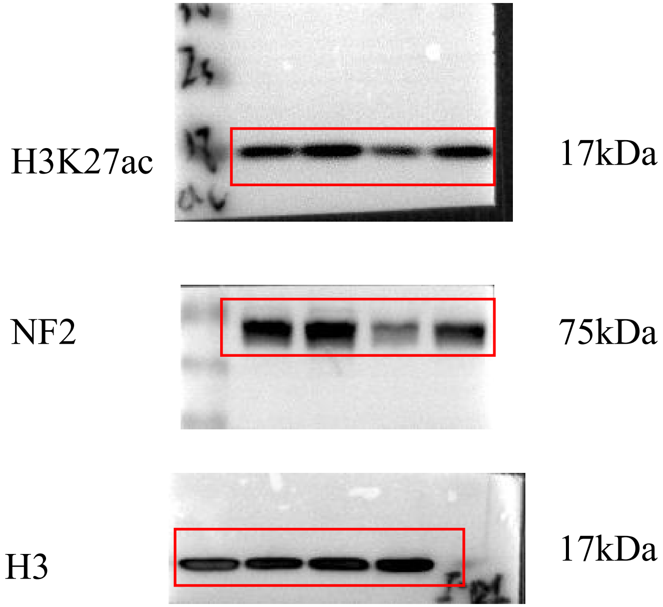

Supplement: Supplementary file 2 — Supporting File 2: advs74199‐sup‐0002‐Data.zip. [file ADVS-13-e22756-s002.zip › Raw Data of Blots.pdf]
